# Single‐Cell Profiling Reveals a Protective WNT5A‐ATF3‐FOSB Signaling Axis in Hair Follicle Stem Cells During Androgenetic Alopecia

**DOI:** 10.1002/advs.77109

**Published:** 2026-08-12

**Authors:** Ruiyu Luo, Ke Fang, Changjiang Zhao, Jiachao Xiong, Yuan Zhu, Mingyang Lu, He Yan, Zihan Li, Baojin Wu, Hua Jiang, Yufei Li

**Affiliations:** ^1^ Department of Plastic Surgery Shanghai East Hospital School of Medicine Tongji University Shanghai China; ^2^ School of Medicine Tongji University Shanghai China; ^3^ St. Hugh's College University of Oxford Oxford UK; ^4^ Department of Plastic Surgery Huashan Hospital Fudan University Shanghai China; ^5^ Shanghai East Hospital Nanjing Medical University Shanghai China

**Keywords:** androgenetic alopecia, ATF3, hair follicle stem cell, single‐cell analysis, WNT5A

## Abstract

Androgenetic alopecia (AGA) is a common form of hair loss with limited treatment options, driven by poorly understood molecular mechanisms. Using single‐cell RNA sequencing of human scalp follicles from balding and non‐balding regions, we constructed a high‐resolution molecular anatomy atlas of the hair follicle and its microenvironment. We identified significant silencing of Wingless‐related integration site 5A (WNT5A) signaling, which was widely known as the trigger of non‐canonical WNT signaling pathway, in hair follicle stem cells (HFSCs) from balding areas, leading to downregulation of the transcription factor ATF3 and its target FOSB. Therapeutically, functional validation showed that activating the WNT5A‐ATF3‐FOSB axis maintained HFSC competence in AGA murine models and significantly inhibited the progression of AGA. Our findings reveal a critical molecular axis in AGA pathogenesis, highlighting its potential as a therapeutic target for hair loss disorders.

## Graphical abstract

Androgenetic alopecia (AGA) is a common form of hair loss with limited treatment options. Silencing of WNT5A signaling, which is widely known as the trigger of the ncWNT signaling pathway, happens in hair follicle stem cells from balding areas. It leads to downregulation of ATF3 and its target FOSB. Activating the WNT5A‐ATF3‐FOSB axis inhibits the progression of AGA in mouse model.

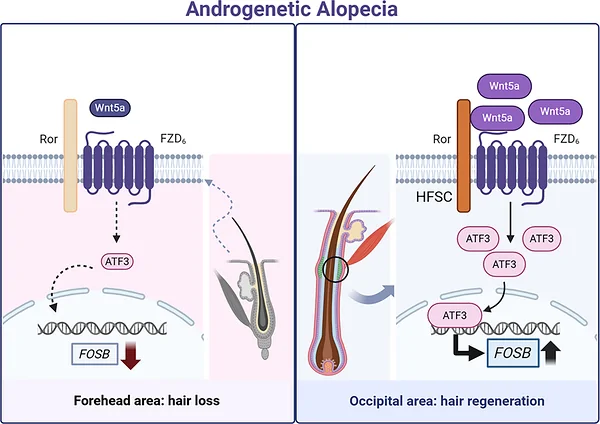

## 1 Introduction

Androgenetic alopecia (AGA), as the leading cause of non‐cicatricial alopecia, has contributed to an increasing disease burden worldwide in recent years [1]. The global prevalence among men is approximately 20%–50%, affecting up to half of Caucasian men over the age of 50 [2]. It is typically characterized by a receding hairline that begins in the frontal and temporal scalp and may progress to the vertex [3]. Beyond its physical manifestations, AGA can significantly impact psychological well‐being, leading to reduced self‐esteem, anxiety, depression, and other emotional concerns [4].

However, the current management of AGA continues to face multiple challenges [5]. Although commonly used pharmacological treatments such as finasteride and minoxidil demonstrate certain efficacy, their effectiveness remains suboptimal and is associated with notable limitations [6, 7]. In particular, anti‐androgen therapies represented by finasteride exhibit considerable heterogeneity in treatment response among patients, suggesting that mechanisms beyond androgen signaling may play significant roles in the pathogenesis of AGA [8]. Despite diverse basic research on hair follicle development and cycling, the precise etiology underlying this common condition remains incompletely elucidated [9, 10, 11]. Moreover, the cellular heterogeneity within hair follicles and the cell type‐specific molecular alterations in hair follicle stem cells (HFSCs) during AGA remain poorly understood. Consequently, further investigation into the underlying pathogenic mechanisms of AGA and the identification of potential therapeutic targets are of critical importance.

As the primary structure involved in hair loss, the hair follicle (HF) possesses a highly complex architecture and microenvironment [12]. The HF cycle progresses through phases of anagen, catagen, and telogen [13]. Mice exhibiting synchronized hair growth waves serve as valuable models for HF research, and studies in mice have elucidated hair lineage dynamics originating from adult stem cells residing in the HF bulge (BG) and hair germ (HG) [14, 15]. To date, much of our understanding of AGA mechanisms derives from mechanistic studies in murine models [16]. However, a detailed molecular atlas of the human hair follicle remains unelucidated, and no study has yet comprehensively explored the specific molecular alterations across distinct cell populations within balding HFs of AGA patients. Identifying key molecular changes in HFs from affected areas is essential for clarifying the underlying pathogenic mechanisms of AGA.

Wnt signaling is a central regulator of hair follicle cycling, with canonical Wnt/β‐catenin signaling promoting HFSC activation and anagen entry [17]. In contrast, the role of non‐canonical Wnt ligands, particularly WNT5A, remains context‐dependent and controversial. While developmental studies suggest that WNT5A contributes to tissue polarity and hair follicle morphogenesis [18, 19], several studies have reported its inhibitory effects on hair growth [20]. Importantly, most previous studies rely on bulk tissue transcriptomics or non‐cell type‐specific perturbation models, which obscure the heterogeneous cellular responses within hair follicles [21, 22]. Only a small number of studies focused on cell heterogeneity in AGA [23]. Therefore, whether WNT5A exerts pro‐ or anti‐regenerative effects in specific hair follicle stem cell populations during AGA remains unresolved.

Here, we employed single‐cell RNA sequencing (scRNA‐seq) to construct a comprehensive molecular atlas of HFs obtained from AGA patients via cosmetic surgery, sampling both balding (forehead) and non‐balding (occipital) regions. By systematically characterizing the gene expression profiles and spatial distributions of distinct cell populations within human HFs, we uncovered unexpected molecular alterations in HFSCs from balding areas: the downregulation of a protective WNT5A‐ATF3‐FOSB signaling axis. Furthermore, we conducted in‐depth functional validation to explore potential mechanisms underlying AGA pathogenesis.

## 2 Results

### 2.1 Construction of a Single‐Cell Transcriptomic Atlas of Human Hair Follicles

To establish a high‐resolution single‐cell transcriptomic atlas of human hair follicles, we obtained informed consent and ethical approval before collecting hair follicle samples during hair transplantation procedures. Follicular unit extraction (FUE) was employed to harvest follicles from both the forehead (balding) and occipital (non‐balding) regions of each patient, enabling paired intra‐individual comparisons. The FUE method minimizes contamination from extraneous skin tissues while preserving the peri‐follicular microenvironment essential for hair follicle function. Subsequently, the isolated HFs were dissociated into single‐cell suspensions and subjected to scRNA‐seq using the 10x Genomics platform.

The Seurat clustering method was utilized to analyze the single‐cell transcriptomic data and robustly assign cell types. Overall, all sequenced cells in hair follicle and its microenvironment were classified into seven major categories: keratinocytes from the permanent portion of the epidermis (EPI), hair follicle cells in the cycling phase of the hair cycle (HFC), resident melanocytes in the skin and hair follicles (ML), immune cells within the microenvironment (IMM), vascular cells (VASC), fibroblasts (FIB), and smooth muscle cells (SMC) (Figure S1A–H).

Through extensive histological and single‐cell literature research, representative marker genes for relevant cell subpopulations were identified. Sub‐clustering of the seven main cell classes yielded 24 distinct transcriptional subpopulations (Figure S1I,J). It is rarely observed that we did not identify well‐studied dermal papilla cells within the FIB category, which may be related to the extraction method of follicular unit extraction. The hair follicle cell population was isolated from the overall cell ensemble and subjected to dimensionality reduction and clustering using the Seurat algorithm. This analysis revealed that hair follicle intrinsic cells can be broadly categorized into outer layer cell groups (*SFRP1* ^+^) and inner layer cell groups (*MSX2* ^+^) (Figure 1A). We first explored keratin genes as potential marker genes and found that *KRT5* and *KRT85* partially represent outer and inner layer cells, respectively, though with limited specificity (Figure 1B). We then selected the most significantly highly variable genes and identified *SFRP1* and *MSX2* as robust markers for outer layer cells and inner/matrix cells, respectively (Figure 1C). Further immunofluorescence staining validated their capability and reliability as markers for distinguishing these cellular subpopulations (Figure 1D).

**FIGURE 1:**
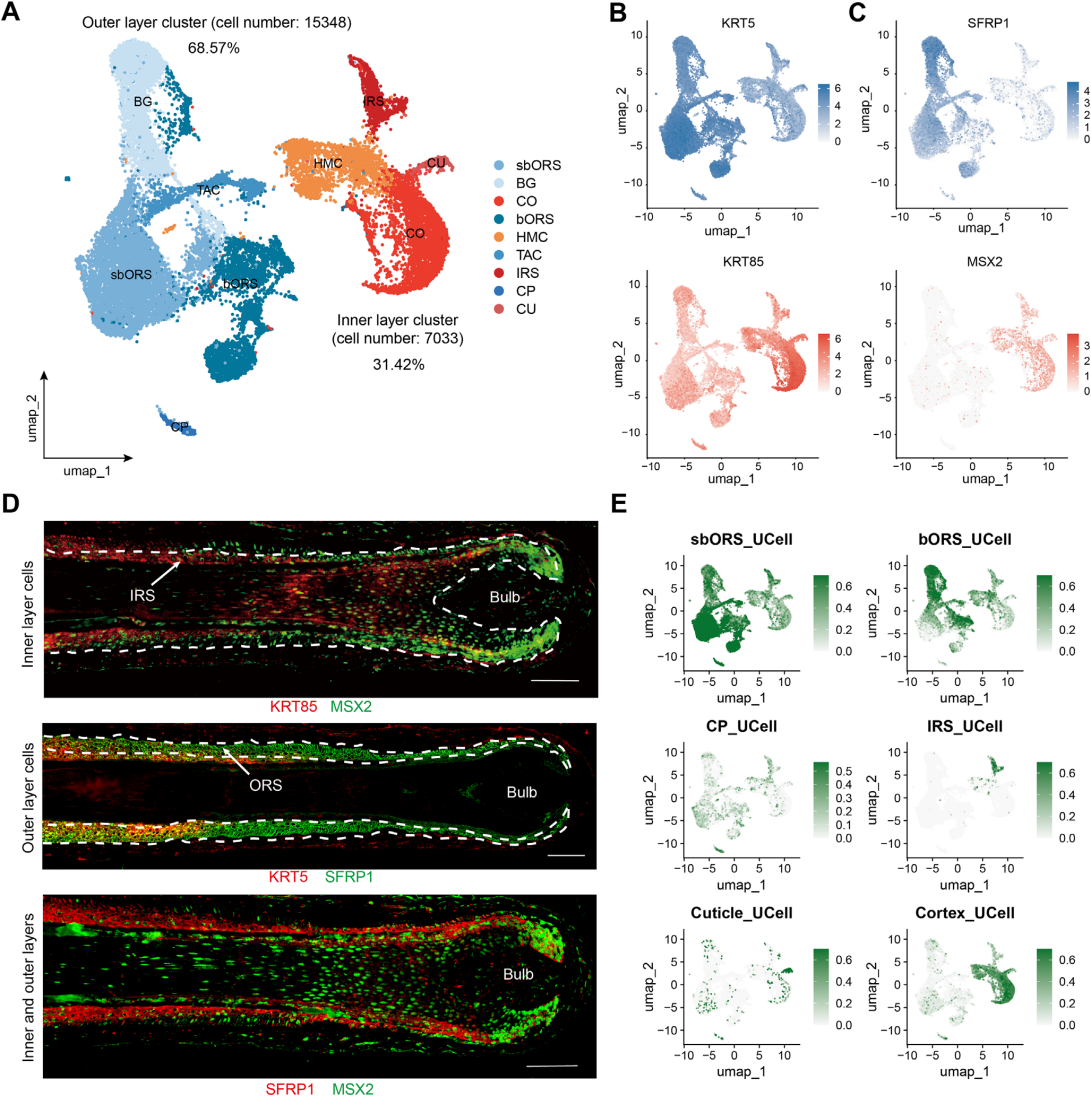
Identification of two main components of hair follicle cells. (A) UMAP projection of single‐cell transcriptomes from human hair follicles. Outer layer cells (*SFRP1* ^+^): BG, bORS, sbORS, TAC. Inner layer cells (*MSX2* ^+^): IRS, CO, CU, HMC. (B) UMAP plots showing expression of representative keratin genes, KRT5 (outer layer) and KRT85 (inner layer). (C) UMAP plots showing expression of the robust marker genes SFRP1 (outer layer) and MSX2 (inner layer). (D) Representative immunofluorescence staining of SFRP1 and MSX2 on longitudinal sections of human anagen hair follicles (*n* = 3, 40×). Scale bars = 100 µm. (E) UCell signature scores for established hair follicle cell subtype gene sets, projected onto the UMAP plot, indicating potential sub‐structures within the major clusters.

Among these, *MSX2* ^+^ inner and matrix cells showed consistency with previous single‐cell and single‐molecule RNA fluorescence in situ hybridization (smRNA‐FISH) studies in mice [24]. In contrast, *SFRP1* was identified as a marker for the outer human HF layers for the first time. A topological branching pattern was observed within both subpopulations, suggesting potential further heterogeneity. UCell scoring based on established marker gene sets for major HF cell subtypes revealed that distinct branches exhibited high scores for gene sets corresponding to different cellular subpopulations (Figure 1E), providing guidance for subsequent sub‐clustering.

### 2.2 Human HF Inner Lineages Originate From Matrix Progenitor Cells

We first analyzed the *MSX2* ^+^ inner and matrix cells of the hair follicle. The clustering results revealed six major cell populations: hair matrix cells (HMC), matrix transit‐amplifying cells (mTAC), inner root sheath cells (IRS), cuticle cells (CU), medulla cells (MED), and cortical cells (CO) (Figure 2A). Among these, the IRS, CU, CO, and MED correspond to the distinct concentric inner layer anatomy of the anagen hair follicle. Spatial localization of MED, CO, CU, and IRS was validated via immunofluorescence staining (Figure 2B). At the chosen resolution, cortical cells were further subdivided into four subpopulations (CO1–CO4). The FindAllMarkers algorithm was applied to identify highly variable genes specific to each cluster within the inner hair follicle compartment, visualized using violin plots (Figure S2). Subsequent cell developmental trajectory and pseudo‐time analyses consistently indicated that hair matrix cells (HMC) serve as the developmental origin of inner layer lineages (Figure S3). In the occipital group, CO cells are the latest differentiated cell group (Figure 2C,D). In the forehead group, IRS cells are the latest differentiated cell group (Figure 2C,D). Located within the matrix, HMCs transition into a matrix transit amplifying cell (mTAC) state before differentiating into various inner layer structural cells, consistent with previous reports [24, 25]. A gene expression heatmap further illustrated the global transcriptional dynamics along this developmental continuum and highlighted stage‐specific marker genes (Figure 2G).

**FIGURE 2:**
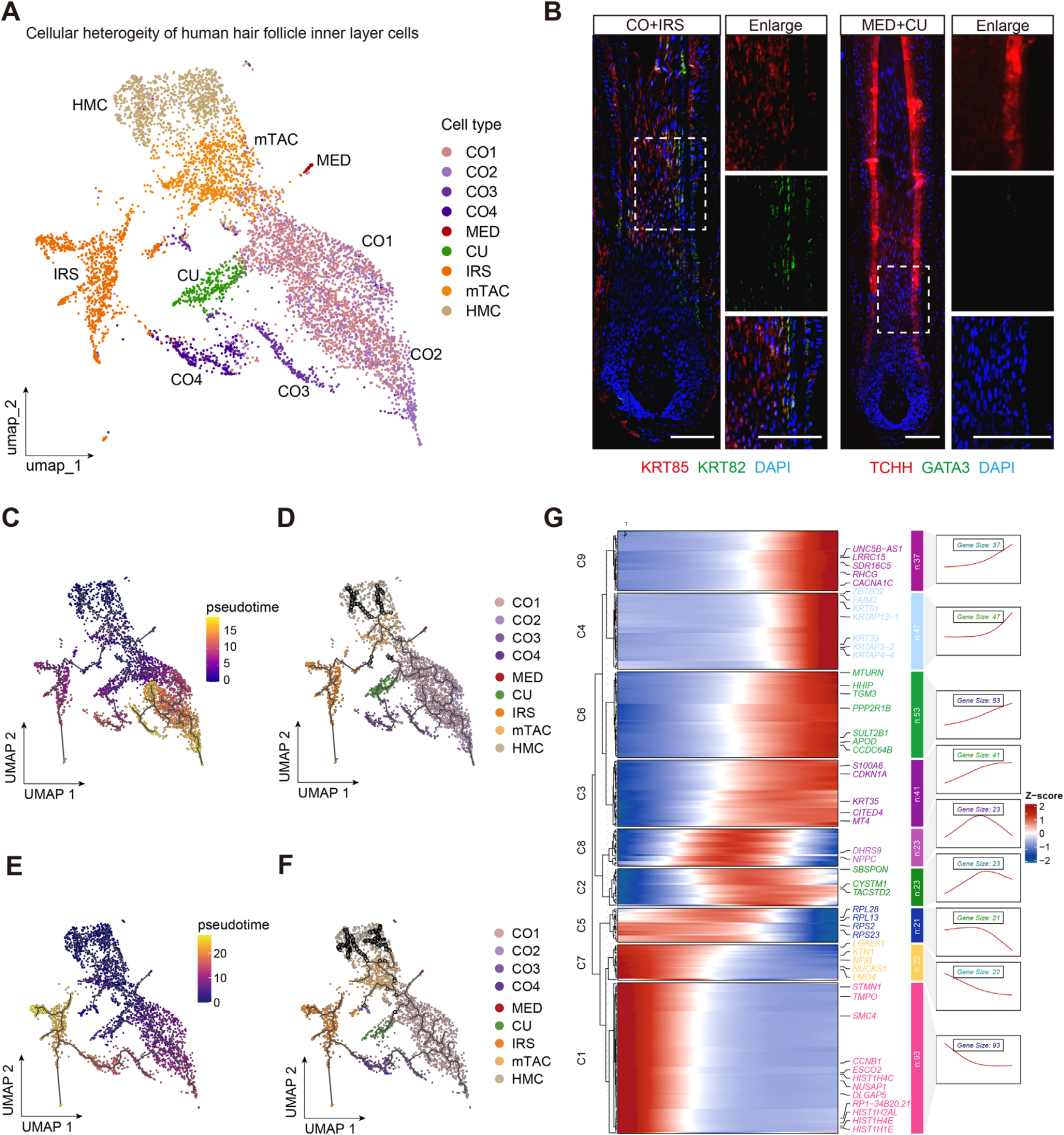
Reconstruction of inner layer differentiation in human hair follicles. (A) UMAP projection of MSX2^+^ inner layer cells, colored by annotated cell types: hair matrix cells (HMC), matrix transit‐amplifying cells (mTAC), inner root sheath (IRS), cuticle (CU), medulla (MED), and cortex cells (CO1‐CO4). (B) Representative immunofluorescence staining for cortical/IRS markers (KRT85/KRT82) and medulla/cuticle markers (TCHH/GATA3) on human hair follicle sections, validating spatial localization. Scale bars, 100 µm. (C and D) UMAP visualization of inner layer cells in the occipital group, colored according to their monocle3 pseudo‐time (C) and cell types (D) as in (A), respectively. (E,F) UMAP visualization of inner layer cells in the forehead group, colored according to their monocle3 pseudo‐time (E) and cell types (F) as in (A), respectively. (G) Heatmap of z‐score normalized expression for highly variable genes across the developmental trajectory from HMCs (left) to terminally differentiated inner layer cells (right).

The topological branching structure revealed that after transitioning into mTACs, HMCs primarily differentiate along three distinct lineages: CU/CO, IRS, and MED. However, whether their fate commitment is predetermined prior to this transition remains unclear. To address this, we further subclustered HMCs and identified four distinct HMC subpopulations (Figure S4A). Among these, subpopulations 2 and 3 exhibited well‐defined marker genes, whereas subpopulations 0 and 1 showed less distinct transcriptional profiles. UCell scoring against marker gene sets of differentiated lineages indicated that subpopulation 1 displayed partial commitment toward CU/CO fate, subpopulation 2 exhibited early IRS characteristics, while subpopulations 0 and 3 showed no significant lineage bias, suggesting they may represent earlier, naiver HMC states (Figure S4B,C). Subsequent developmental trajectory analysis confirmed that subpopulation 3 likely serves as the differentiation origin of HMCs, supporting the conclusions drawn from UCell scoring (Figure S4D–F).

### 2.3 Spatial and Functional Characterization of HF Outer Layer Subpopulations

Sub‐clustering of *SFRP1*‐positive outer layer cells identified five cellular subpopulations: basal outer root sheath cells (bORS), suprabasal outer root sheath cells (sbORS), companion layer cells (CP), basal transit‐amplifying cells (bTAC), and bulge cells (BG). The bORS and sbORS subpopulations were further subdivided into four and two distinct clusters, respectively. Analysis of highly variable genes enabled precise annotation based on significantly enriched marker genes and functional enrichment results. Specifically, the four bORS subpopulations were functionally annotated as follows: Wnt signaling inhibition (bORS‐WI‐SERPINF1), extracellular matrix construction (bORS‐ECM‐COL4A1), participation in skin development (bORS‐SD‐TNFRSF19), and terminal differentiation of hair follicles (bORS‐TD‐PPARGC1A). The two sbORS subpopulations were designated to roles in keratinization (sbORS‐KR‐KRT6B) and tissue polarity establishment (sbORS‐PL‐PARD3) (Figure 3A–E). Spatial localization of all six ORS subpopulations was confirmed via immunofluorescence staining (Figure S5A–F).

**FIGURE 3:**
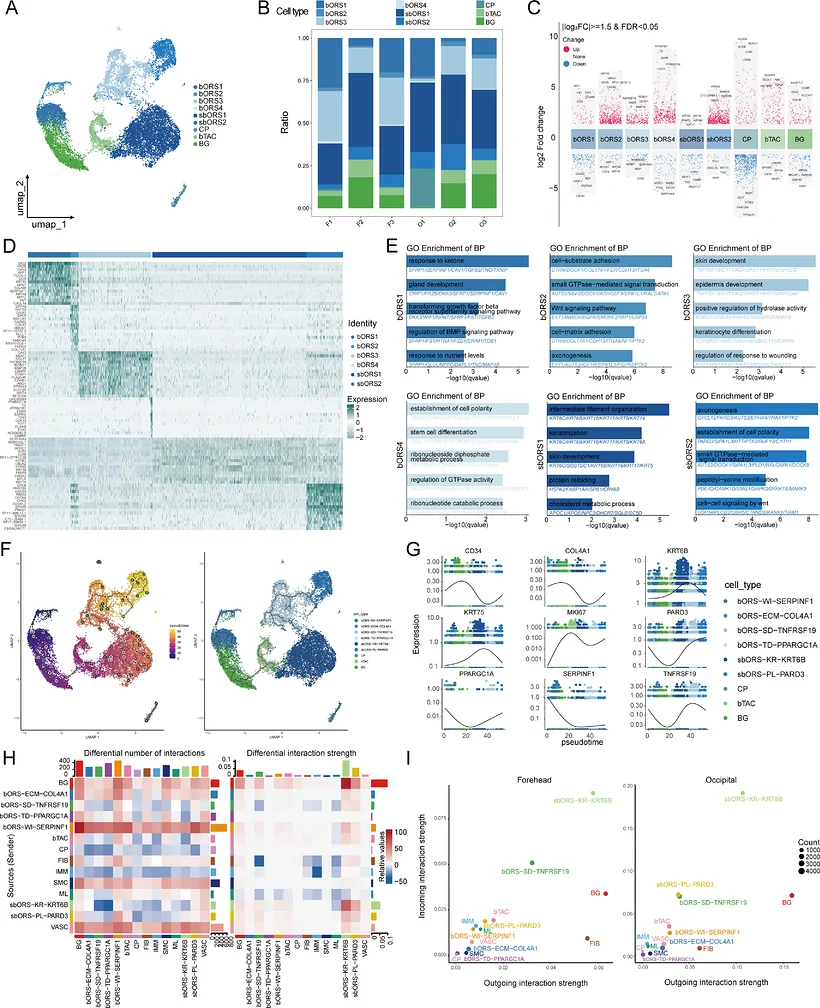
Single cell analysis revealing the construction and cellular interaction of outer layer cells. (A) UMAP projection of SFRP1^+^ outer layer cells, colored by annotated cell types: bulge (BG), companion layer (CP), basal transit‐amplifying cells (bTAC), and subpopulations of basal outer root sheath (bORS) and suprabasal outer root sheath (sbORS). (B) Stacked bar plot showing the proportional composition of each outer layer cell type from individual patient samples (*n* = 3 patients). (C) Volcano plot showing high variable genes of different cell clusters. (D) Heatmap of average z‐score normalized expression for the top marker genes defining each outer layer cell subpopulation. (E) GO enrichment analysis annotated each cell type with its cellular function. (F) UMAP visualization of outer layer cells, colored according to their monocle3 pseudo‐time (left) and cell types (right), respectively. (G) Different cell type markers’ expression level among all cells, revealing a consistency in development. Specifically, *CD34* for BG, *COL4A1* for bORS‐ECM‐COL4A1, *KRT6B* for sbORS‐KR‐KRT6B, *KRT75* for CP, *MKI67* for bTAC, *PARD3* for sbORS‐PL‐PARD3, *PPARGC1A* for bORS‐TD‐PPAGC1A, *SERPINF1* for bORS‐WI‐SERPINF1, *TNFRSF19* for bORS‐SD‐TNFRSF19. (H) Differential number of interactions between outer layer cells and microenvironmental cells in occipital (non‐balding) versus forehead (balding) follicles, analyzed by CellChat. Red, increased in occipital; blue, increased in forehead. (I) Dot plot summarizing the changes in incoming and outgoing interaction strengths for each outer layer cell type between occipital and forehead regions.

Based on our histological knowledge, *KRT15* ^+^ *CD34* ^+^ bulge cells are defined as hair follicle stem cells (HFSC) and serve as the developmental origin of outer layer cells. Pseudo‐time analysis revealed two distinct differentiation trajectories of these stem cells: one directly differentiating into bORS‐WI‐SERPINF1 and bORS‐ECM‐COL4A1 subpopulations, and the other first transitioning into transit‐amplifying cells before further differentiating into bORS‐SD‐TNFRSF19 and both sbORS subpopulations. In contrast, companion layer cells and the terminally differentiated bORS‐TD‐PPARGC1A subpopulation did not exhibit clear developmental relationships with HFSCs in the computational trajectory inference (Figure 3F,G). Moreover, such a differentiation process is possibly the same both in forehead group and occipital group, revealing that there may be no obvious barrier happens in outer cell differentiation in AGA environment (Figure S6A–F).

Cell proportion analysis didn't reveal significant differences in cell type ratio between occipital healthy tissue and forehead alopecia tissue, demonstrating that the main cause to AGA may not be cell type change, but possibly be deeper reason at molecular level (Figure S7A,B). In the initial clustering, in addition to intrinsic hair follicle cells, a substantial number of interfollicular epidermal cells (IFE) and stromal cells were identified. Given that the majority of the hair follicle is embedded within the dermis, we focused on the interaction between stromal cells and outer layer hair follicle cells. Using the CellChat algorithm, we then observed that the number and strength of interactions among outer layer cells and their microenvironment were more abundant in occipital follicles compared to frontal follicles (Figure 3H and Figure S8A). It was observed that several pathways, including EPHB, NOTCH, DESMOSOME, THBS and COLLAGEN signaling pathways were stronger in the occipital healthy region (Figure S9A). Circle plots further illustrated the strength of representative signaling interaction between different cell types (Figure S10A–J). Interestingly, the Wnt signaling pathway, which was widely known as a trigger of hair growth, was upregulated in balding region (Figure S10J), showing the complexity of cellular signals in AGA. Specifically, the BG, bORS‐WI‐SERPINF1, smooth muscle cells (SMC), and vascular endothelial cells (VASC) in the occipital region exhibited higher signal output in terms of both quantity and intensity. Moreover, BG and sbORS cells in the occipital region received significantly stronger and more numerous signals (Figure 3H,I). Given the consistency in signal input and output, it was reasonable to conclude that the HFSC population represented by BG undergoes significant signal attenuation in the frontal region during the hair loss process. After excluding signaling pathways such as CD22 and CD23 signaling pathways, which were largely unexpressed in hair follicle‐related cells, we identified globally top weakened signaling pathways in the frontal region. Notably, WNT5A within the non‐canonical WNT (ncWNT) pathway was significantly downregulated in BG cells (Figure S10A–H). We also checked WNT5A expression mode in scRNA‐seq results and found it's widely expressed by hair follicle cells, especially by HFSCs, HMC, and TAC (Figure S11I,J). But back to the violin plot, its's easy to find that only HFSCs in forehead region displayed a reduced expression of WNT5A. We would further explore this finding in the following section.

### 2.4 HFSCs in the Balding Region Exhibit Silenced WNT5A Signaling

Based on the above analysis, we first performed further analysis on the BG, namely the bulge hair follicle stem cells. To make sure that KRT15 is a robust marker for HFSCs in the bulge region, we first did IF staining of KRT15, LGR5, and SOX9 in hair follicle tissue. The result showed that KRT15 was reliable for HFSC marking (Figure S12A). Then we did further analysis in scRNA‐seq data. At a certain resolution, the algorithm divided the hair follicle stem cells into three clusters (Cluster 0, Cluster 1, and Cluster 2). GO enrichment analysis and KEGG enrichment analysis annotated the representative functions of each cluster (Figure 4A,B). Cluster 0 (*LMCD1* ^+^) was primarily associated with cytoskeleton construction, Cluster 1 (*MGST1* ^+^) with transmembrane transport, and Cluster 2 (*KRT7* ^+^) with interactions with the extracellular matrix. In the UMAP projection, Cluster 0 was well separated from Clusters 1 and 2, while Clusters 1 and 2 were intermixed, suggesting potential biological similarity between them (Figure 4C and Figure S12B). Further immunofluorescence staining illustrated the spatial distribution of these three cell populations within the hair follicle (Figure 4D). Given our previous finding that hair follicle stem cells exhibit significant signaling differences between the frontal and occipital regions of AGA patients, we employed a pseudo‐bulk approach to analyze differentially expressed genes in the hair follicle stem cell population. A volcano plot revealed a substantial number of genes downregulated in the frontal region compared to the occipital region, consistent with attenuated signaling patterns (Figure 4E). GSVA scoring of 50 hallmarks GSEA pathways also demonstrated widespread downregulation of pathways in frontal hair follicle stem cells (Figure 4F).

**FIGURE 4:**
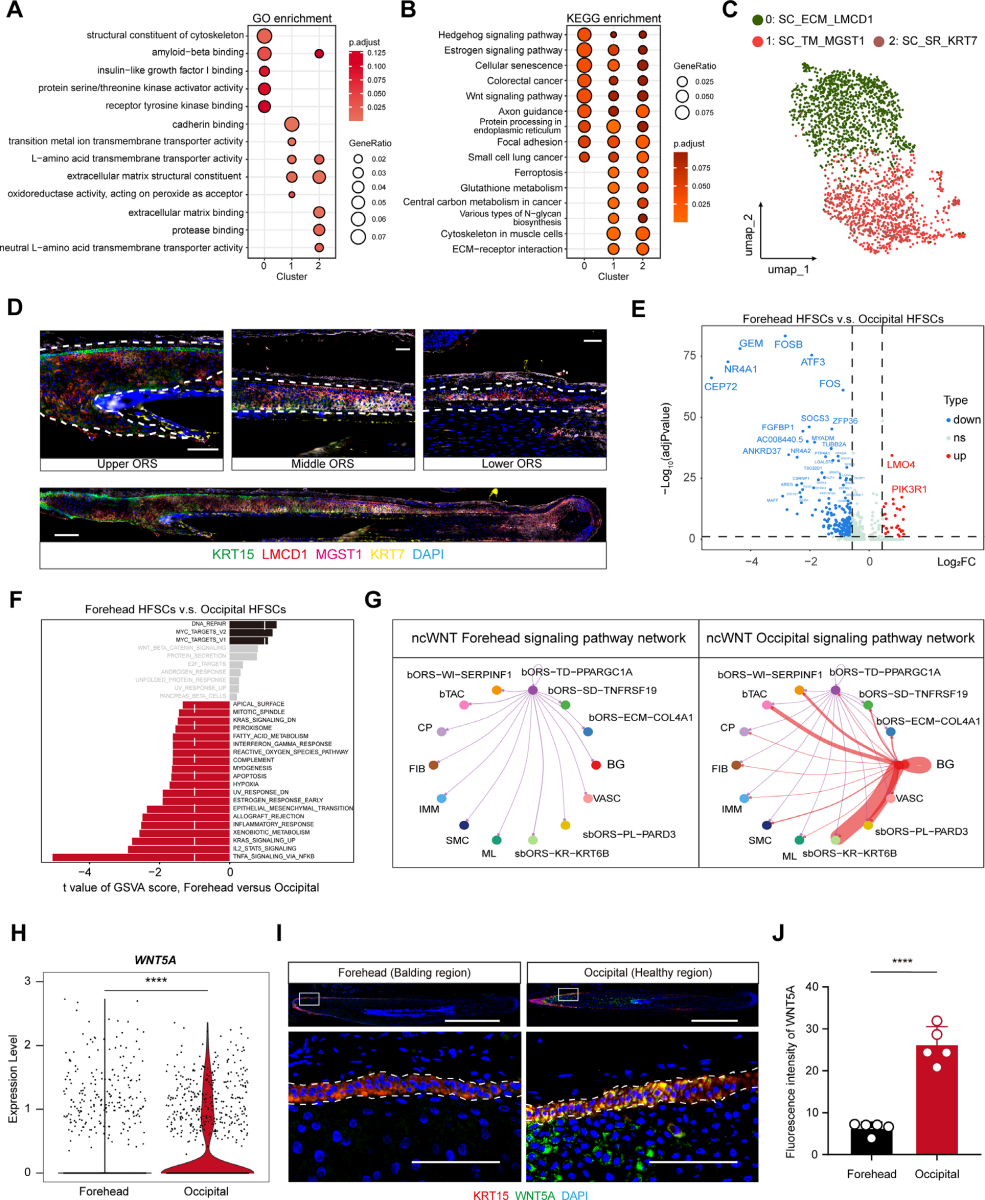
Discovery of inhibited WNT5A expression in forehead HF via analysis of HFSCs. (A and B) Select GO biological process terms (A) and KEGG pathways (B) enriched in the three HFSC subclusters. (C) Relative distance between three clusters on UMAP plot. (D) Immunofluorescence staining of clusters’ markers (KRT15 for all HFSCs, LMCD1 for SC‐ECM‐LMCD1, MGST1 for SC‐TM‐MGST1, KRT7 for SC‐SR‐KRT7). Scale bar = 100 µm. (E) Volcano plot of differentially expressed genes in HFSCs from forehead (balding) versus occipital (non‐balding) regions (*n* = 3 patients). Genes with log_2_(fold change) > 1.5 and adjusted p‐value < 0.05 are highlighted in red (downregulated in occipital region) and blue (upregulated in occipital region). (F) Gene Set Variation Analysis (GSVA) scores for the Hallmark gene sets in HFSCs from frontal and occipital regions (*n* = 3). (G) CellChat analysis showing the significantly weakened outgoing non‐canonical WNT (ncWNT) signaling from bulge (BG) HFSCs in the frontal region compared to the occipital region. (H) Violin plot further showed inhibited WNT5A expression in forehead HFSCs. (I,J) Immunofluorescence staining against WNT5A and HFSC marker KRT15 confirmed the result of bioinformatic analysis (*n* = 5 hair follicles per group, 3 random fields per follicle) (I) and the quantification of fluorescence intensity (J). Upper scale bar = 500 µm, lower scale bar = 100 µm. Data are presented as mean ± SD. ^****^: *p* < 0.0001, determined by unpaired Students’ *t*‐test.

Regarding the previously identified ncWNT pathway, we examined its specific alterations in the network analysis and observed that BG cells in the frontal region do not emit much ncWNT signals, whereas this signaling activity is highly robust in the occipital region (Figure 4G). We further assessed the expression patterns of WNT5A using violin plots, which revealed a significantly higher proportion of WNT5A‐positive BG cells in the occipital region compared to the frontal region (Figure 4H). Comparative immunofluorescence staining of patient samples confirmed the silencing of WNT5A signaling in the bulge area during hair loss (Figure 4I).

Although previous bulk transcriptomic studies have suggested downregulation of the canonical WNT pathway in hair follicles of AGA patients, and activation of WNT signaling in HFSCs has been shown to promote hair growth [22, 26], our findings indicate that silencing of the non‐canonical WNT pathway within bulge HFSCs, rather than dysregulation of the canonical WNT pathway, may represent a key mechanism underlying AGA pathogenesis.

### 2.5 WNT5A Activates ATF3 Expression in HFSCs and Inhibits AGA Progression

As an initiator of the ncWNT pathway, WNT5A is secreted by cells in the bulge region and acts on neighboring cells, yet it may not directly execute hair regeneration functions. We therefore hypothesized that some of the differentially expressed genes identified computationally may result from WNT5A signaling differences and play critical roles in AGA pathogenesis. Surprisingly, the most significantly altered genes were predominantly transcription factors, including *FOS*, *FOSB*, *GEM*, and *ATF3* (Figure 4E). We subsequently performed GSEA analysis using transcription factor gene sets on these downregulated genes in HFSCs from balding regions. The results revealed that the gene set associated with *ATF3* was the most significantly enriched (Figure 5A). To investigate whether ATF3 acts as a functional downstream effector of WNT5A signaling in HFSCs, we conducted a series of validation experiments. Transcriptomic analysis and immunofluorescence staining of patient samples consistently demonstrated significantly reduced ATF3 expression in balding regions, with its expression specifically localized to *KRT15* ^+^ HFSCs (Figure 5B–D). In vivo molecular dynamics experiments showed that *ATF3* expression in HFSCs increased in a time‐dependent manner in mouse skin treated with Foxy‐5 (a WNT5A agonist) following depilation (Figure 5E,F). Similarly, in vitro experiments confirmed that Foxy‐5 treatment significantly upregulated ATF3 expression in HFSCs (Figure 5G–I). These findings collectively indicate that WNT5A signaling promotes ATF3 expression in HFSCs.

**FIGURE 5:**
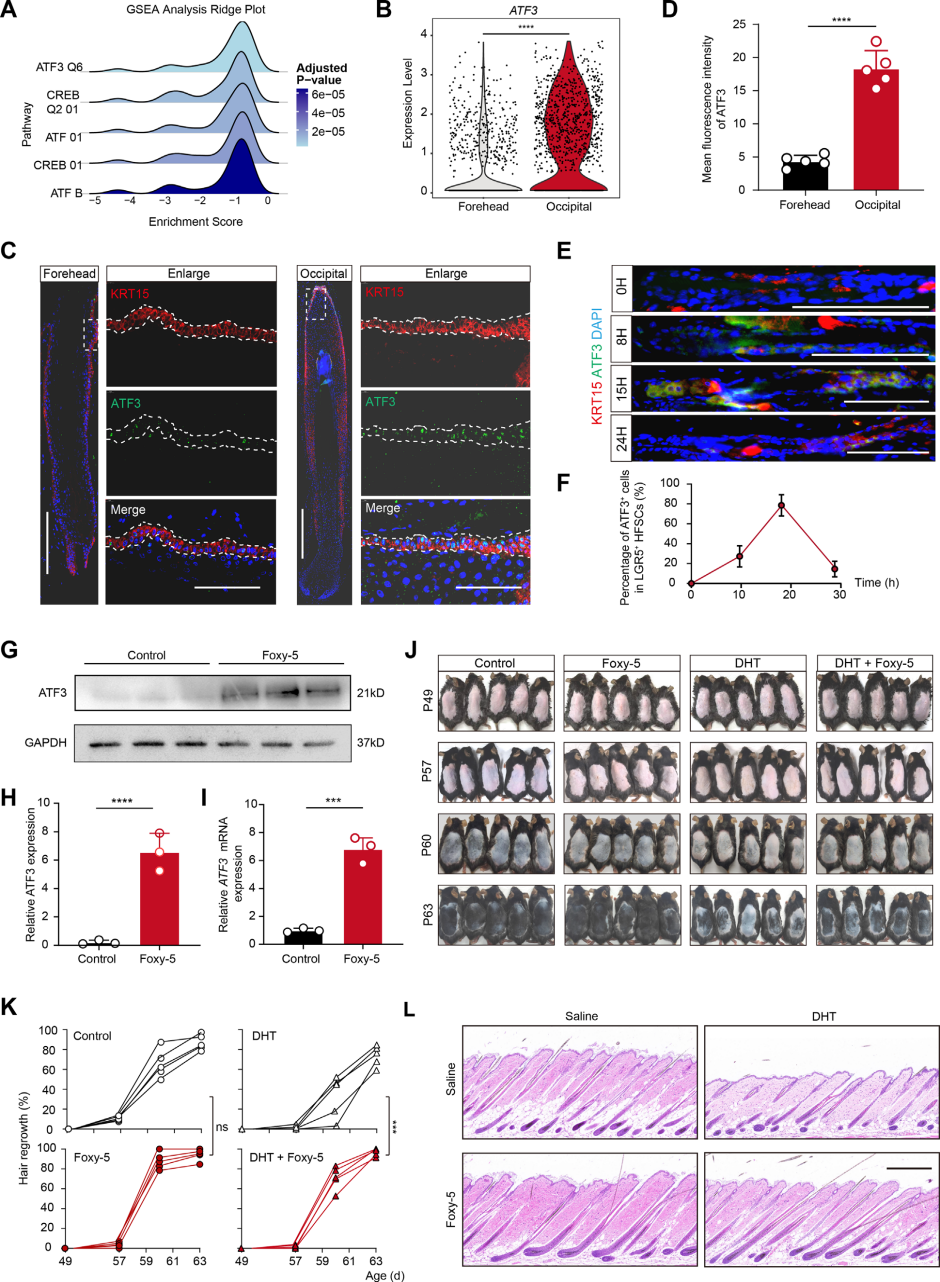
WNT5A activation promotes the expression of ATF3 and resists AGA progression. (A) GSEA analysis based on TF gene sets displayed by ridge plot. (B) Violin plot revealing down‐regulated *ATF3* level in the forehead balding area. (C,D) Representative images of immunofluorescence staining against ATF3 confirmed results from bioinformatic analysis (C), and their quantitative results (D) (*n* = 5). Left scale bar = 500 µm; right scale bar = 100 µm. (E,F) Immunofluorescence staining showing the activation of ATF3 after Foxy‐5 treatment (E), quantification was conducted via calculating the number of ATF3+LGR5+ HFSCs per view (F) (*n* = 3). Scale bar = 100 µm. (G,H) Western blot of ATF3 after activation of WNT5A (G) and its quantification (H) (*n* = 3). (I) WNT5A activation up‐regulated the transcriptional level of *ATF3* (*n* = 3). (J,K) Images (J) and quantification (K) of hair regrowth after WNT5A activation via Foxy‐5 treatment (*n* = 5). (L) H&E staining sections from control mice injected with Foxy‐5, followed by DHT treatment (*n* = 5). Scale bar = 500 µm. Data are presented as mean ± SD. Ns: not significant, ^*^ *p* < 0.05, ^***^ *p* < 0.001, ^****^ *p* < 0.0001, determined by unpaired Students’ *t*‐test.

Given the observed attenuation of WNT5A signaling during hair loss, we conducted WNT5A agonism experiments in the AGA mouse model to determine whether activation of WNT5A signaling could inhibit AGA progression and promote hair regeneration. Notably, we found that Foxy‐5 (a WNT5A agonist) treatment significantly mitigated the delayed hair growth induced by AGA modeling, demonstrating measurable hair‐promoting effects, including accelerated hair regeneration in depilated areas, increased hair bulb size, and enhanced skin thickness (Figure 5J–L and Figure S13A,B). Furthermore, Foxy‐5 administration led to elevated regenerative capacity of HFSC‐derived hair matrix cells (Figure S13C,D). Moreover, we used an inhibitor of WNT5A signaling, Box5, to see whether the inhibition of WNT5A could also affect hair growth. Surprisingly, Box5 significantly inhibited hair regrowth in AGA mouse model, again verifying the importance of WNT5A signaling in resisting AGA pathogenesis (Figure S14A–D).

In summary, aberrant silencing of WNT5A represents a potential underlying mechanism driving AGA pathogenesis. Both in vivo and in vitro experiments demonstrate that WNT5A signaling likely ameliorates the hair loss phenotype in AGA by enhancing ATF3 expression in HFSCs, thereby promoting hair regeneration.

### 2.6 WNT5A–ATF3–FOSB Axis Plays a Role in HFSCs

As a classical stress‐responsive transcription factor, how ATF3 acts downstream to exert its anti‐alopecia biological effects remains unclear. To investigate the potential mechanisms mediated by ATF3 in HFSCs, we performed cleavage under targets & tagmentation (CUT&Tag) assays. Genome‐wide analysis identified 22,146 ATF3 binding peaks, among which 19.48% were located in promoter regions of target genes (Figure 6A,B). GO and KEGG enrichment analyses of these promoter‐associated genes revealed significant enrichment of terms related to cell morphogenesis and development (Figure 6C,D). Meanwhile, basing on prior GSEA results of differentially expressed genes, we constructed a network map of the top five enriched pathways and identified FOSB, GEM, AREG, and RCAN1 as centrally positioned genes (Figure 6E). Considering gene expression levels, we observed that FOSB was notably downregulated in balding regions. Using the JASPAR database for transcription factor binding site prediction, we identified high‐confidence ATF3 binding sites (score > 10) in the promoter regions of FOSB in both humans and mice (Figure 6F). Examination of ATF3 binding peak distribution on FOSB confirmed significant binding within the promoter region (1–2 kb upstream) (Figure 6G). ChIP‐PCR and dual‐luciferase reporter assays further demonstrated that ATF3 binds to the FOSB promoter and enhances its transcription in HFSCs (Figure 6H,I). We then treated mouse primary HFSCs with an ATF3 agonist in vitro and validated that ATF3 upregulates FOSB expression via Western blot and qPCR (Figure 6J,K). Finally, we confirmed reduced FOSB expression and fewer FOSB‐positive cells in human balding hair follicles, consistent with single‐cell expression data (Figure 6L–N). These results demonstrate that ATF3 promotes FOSB expression, thereby delineating a potential WNT5A–ATF3–FOSB axis that may inhibit AGA progression.

**FIGURE 6:**
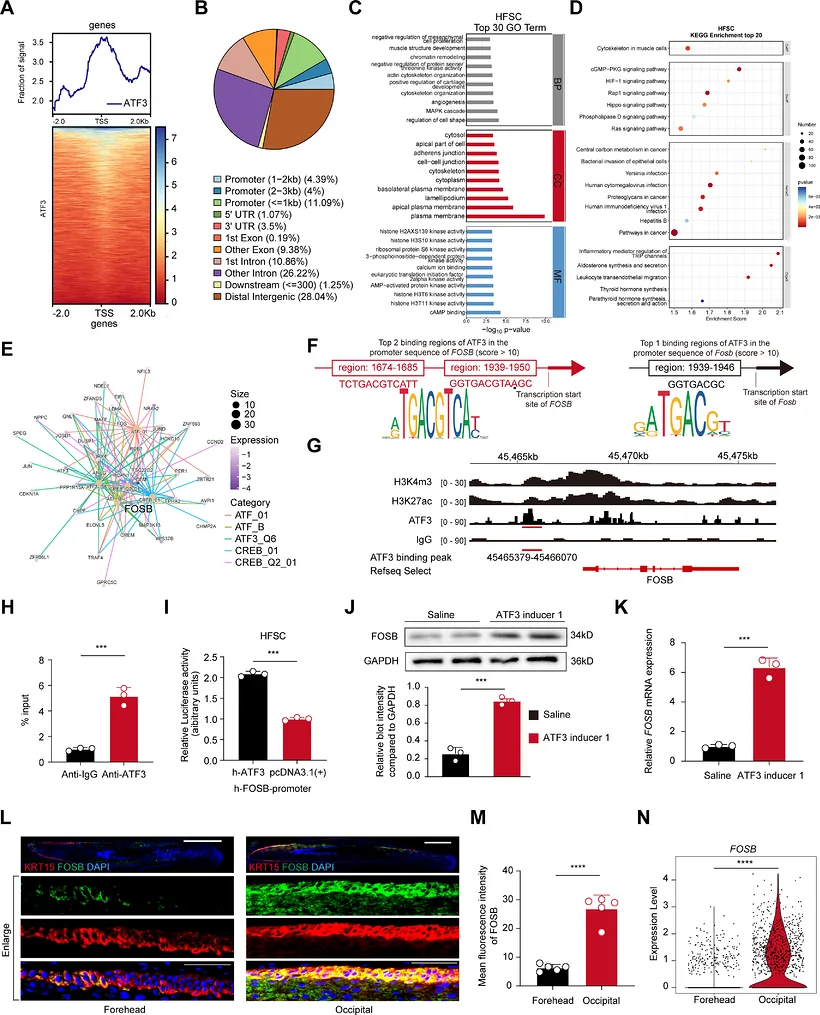
FOSB is the robust target of ATF3, whose expression is inhibited in HFSCs of balding hair follicles. (A) Heatmap analysis of ATF3 signals near TSS regions. (B) Pie chart demonstrating the annotation of peaks’ binding regions. (C) GO analysis of target genes of ATF3 in HFSCs. (D) KEGG analysis of target genes of ATF3 in HFSCs. (E) Network showing relationship between key genes and significantly enriched terms from DEGs in HFSCs. (F) Putative ATF3‐binding sites identified in the promoter region of *FOSB*/*Fosb* gene via JASPAR tool. (G) IGV tracks showing ATF3 binding on *FOSB* gene's promoter in HFSCs. (H) Chromatin immunoprecipitation (ChIP) coupled with PCR provided evidence of ATF3 binding to the promoter region of *FOSB* gene (n = 3). (I) Dual‐luciferase reporter assay in HFSCs co‐transfected with a FOSB promoter‐driven firefly luciferase construct and an ATF3 expression vector or empty control (*n* = 3 independent transfections). (J and K) Activation of ATF3 also promoted expression of FOSB, proved by Western blot (J) and RT‐qPCR (K) (for Western blot, *n* = 3 with 2 replicates in one blot; for PCR, *n* = 3). (L,M) Significant decrease of the expression of FOSB in HFs in forehead balding area (L) and the quantification of fluorescence intensity (M) (*n* = 5). Upper scale bar = 1000 µm, lower scale bar = 100 µm. (N) Violin plot showing decreased transcriptional level of FOSB in HFSCs detected by scRNA‐seq. Data are presented as mean ± SD. ^***^ *p* < 0.001, ^****^ *p* < 0.0001, determined by paired (only in Figure 6I) and unpaired Students’ *t*‐test.

### 2.7 ATF3 Promotes Hair Growth and Mediates the Anti‐AGA Effect of WNT5A

After identifying the potential downstream targets of WNT5A, we conducted a series of validation experiments targeting ATF3 and FOSB separately. As a validated downstream effector of WNT5A, we hypothesized that ATF3 alone could inhibit the AGA phenotype. Treatment with ATF3 inducer 1, which was a compound previously confirmed to effectively upregulate ATF3 expression, produced effects similar to WNT5A agonism in mice: ATF3 activation counteracted DHT‐induced inhibition and promoted hair regrowth (Figure 7A). In non‐pathological states, mice treated with ATF3 inducer 1 exhibited higher hair density at the experimental endpoint. In DHT‐induced AGA model mice, treatment attenuated DHT‐mediated hair growth delay and even restored hair regeneration rates to near‐normal levels (Figure 7B). Histological analysis revealed that ATF3 inducer 1 treatment significantly increased both the thickness of the anagen‐associated subcutaneous layer and the diameter of hair bulbs (Figure 7C–E). Notably, additional immunofluorescence staining confirmed that ATF3 activation upregulated FOSB expression in LGR5^+^ HFSCs in vivo, further supporting the existence of the WNT5A–ATF3–FOSB molecular axis in its downstream segment (Figure 7F–H). Moreover, rescue experiments further proved that the WNT5A signal activated FOSB via ATF3 (Figure S15A–C).

**FIGURE 7:**
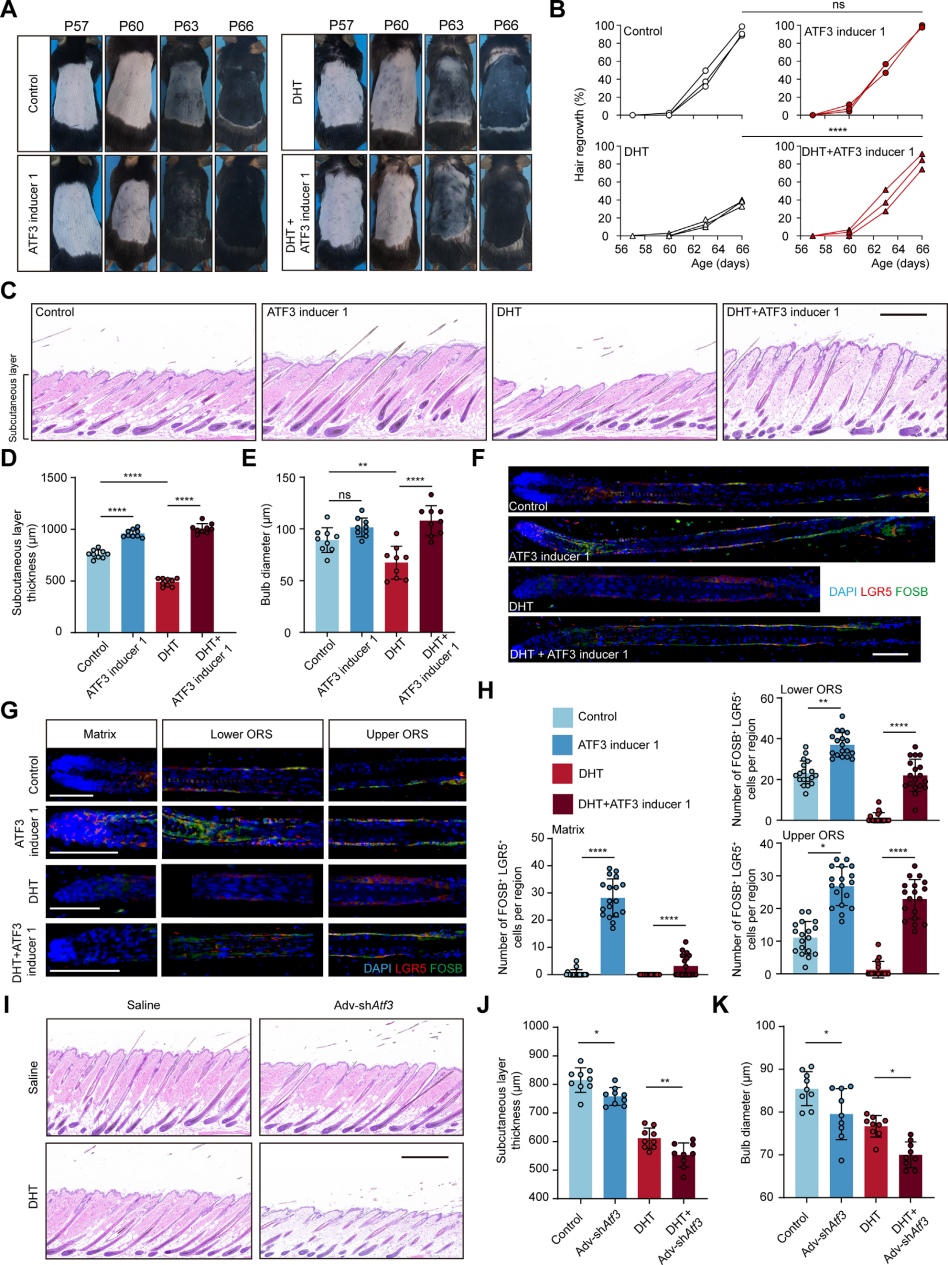
Activion of ATF3 promotes the expression of FOSB and hair regeneration. (A,B) Images (A) and quantification (B) of hair regrowth in AGA mice (*n* = 3). (C) H&E staining of sections from mice skin, demonstrating state of hair regrowth (*n* = 3). Scale bar = 500 µm. (D,E) Bar plots showing the quantification results of subcutaneous layer (the distance from basal layer to the bottom of hair bulb) (D) and hair bulb diameter (E) (*n* = 3, 3 views per sample were selected for the quantification). (F–H) Immunofluorescence staining against FOSB (F,G) and its quantification (H) proving that the activation of ATF3 promoted the expression of FOSB (*n* = 3, 3 views per sample were selected for quantification). Scale bar = 100 µm. (I) H&E staining sections from AGA model and *Atf3*‐knocked‐down mice. Scale bar = 500 µm. (J,K) Hair regrowth quantification by subcutaneous layer thickness (J) and bulb diameter (K) (*n* = 3, 3 random views from per section). Data are presented as mean ± SD. ^*^ *p* < 0.05, ^**^ *p* < 0.01, ^***^ *p* < 0.001, ^****^ *p* < 0.0001, ns: not significant, determined by one‐way ANOVA with Tukey's post hoc test.

To determine whether the anti‐AGA function of WNT5A in HFSCs depends on ATF3, we used adenoviral injection to suppress ATF3 expression in mouse skin. Based on prior immunofluorescence localization results, ATF3 expression is highly specific to HFSCs, enabling relatively targeted modulation of ATF3 levels via adenovirus‐mediated knockdown. Knockdown of Atf3 made alopecia more severe in mice treated with DHT (Figure 7I–K and Figure S16A,B). In vivo knockdown experiments demonstrated that reduced ATF3 expression significantly diminished the hair‐promoting effects of WNT5A, confirming that the anti‐AGA activity of WNT5A is dependent on ATF3 (Figure S17A–E).

### 2.8 FOSB Inhibits AGA Progression by Unleashing the Proliferative Potential of HFSCs

FOSB was recognized as a key regulator of cell proliferation and differentiation [27]. After identifying FOSB as a functional downstream target of ATF3, we further investigated its role in hair growth. Using adenovirus‐mediated transfection, we successfully upregulated FOSB expression in mouse skin tissues (Figure 8A). Consistent with our hypothesis, mice overexpressing *Fosb* exhibited accelerated hair regeneration rates. Although the duration of the telogen phase remained unchanged, hair regeneration progressed more rapidly upon entering anagen (Figure 8B,C). Additionally, increases in hair bulb size and subcutaneous layer thickness were observed (Figure 8D,E). These findings suggest that FOSB levels may modulate the proliferation rate of HFSCs. To quantify the proliferation of HFSCs or their lineage‐derived cells, we performed Ki67 staining on skin tissues at the experimental endpoint. Immunofluorescence results clearly demonstrated that *Fosb* overexpression significantly increased the number of activated HFSCs, indicating that the WNT5A–ATF3–FOSB signaling axis ultimately targets HFSC proliferation and differentiation (Figure 8F). Surprisingly, adenovirus‐mediated knockdown of *Fosb* did not produce statistically significant changes in hair growth under physiological conditions. However, under DHT‐induced stress, *Fosb* knockdown exacerbated AGA progression, suggesting that FOSB itself may serve as an important factor in resisting hair loss (Figure 8G–I and Figure S18A,B).

**FIGURE 8:**
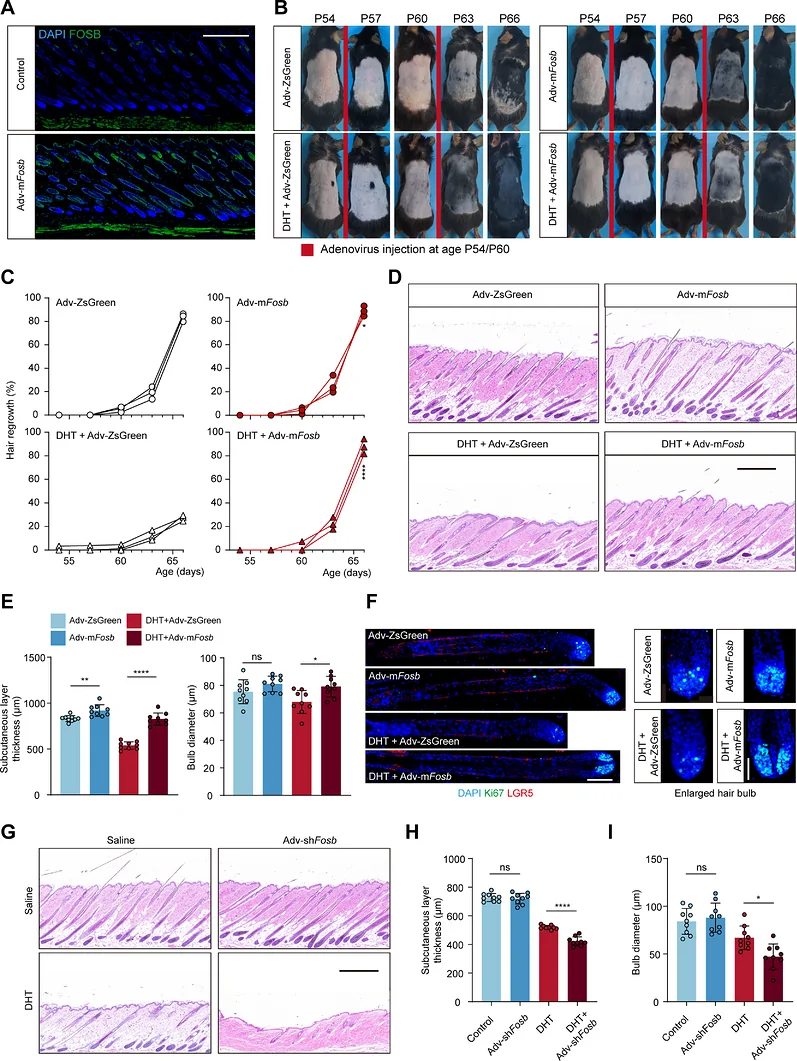
FOSB is the activator of hair regrowth, performing the role of anti‐AGA. (A) Identification of the over‐expression effect via immunofluorescence staining. Scale bar = 500 µm. (B,C) Time‐course images of hair regrowth post‐adenovirus‐injection (B) and the quantification of hair regrowth area's percentage (C) (*n* = 3). (D,E) H&E‐stained sections (D) and hair growth quantification. Scale bar = 500 µm. (E) (*n* = 3, 3 views per sample were selected for the quantification). (F) Representative immunofluorescence staining for Ki67 and LGR5 in mouse skin sections from Adv‐ZsGreen and Adv‐*Fosb* treated groups. Scale bars = 100 µm. Right graph shows quantification of Ki67^+^ positive cells in hair matrix per field of view (*n* = 3, 3 fields per mouse). Scale bar = 100 µm. (G–I) Sections (G) and quantification of hair regrowth (H,I) from *Fosb* knock‐down mice proving the protective role of Fosb in the progression of AGA (*n* = 3, 3 views per sample were selected for the quantification). Scale bar = 500 µm. Data are presented as mean ± SD. ^*^ *p* < 0.05, ^**^ *p* < 0.01, ^***^ *p* < 0.001, ^****^ *p* < 0.0001, ns: not significant, determined by one‐way ANOVA with Tukey's post hoc test.

## 3 Discussion

Employing an integrated approach encompassing scRNA‐seq, CUT&Tag, primary HFSC cultures, and in vivo experiments in AGA mouse models, we constructed a single‐cell atlas of both healthy scalp hair follicles and those in the pathological state of AGA. Our findings demonstrate that downregulation of the WNT5A–ATF3–FOSB axis is a critical factor in AGA pathogenesis, while also confirming that the restoration of this axis is associated with resistance to AGA progression. While the biology of hair follicles has been extensively characterized in mice, the molecular profile of human hair follicles, particularly in the context of androgenetic alopecia, remains poorly elucidated. Prior studies have established comprehensive atlases of murine skin and hair follicles and conducted single‐cell sequencing in AGA mouse models [24, 28]. By leveraging hair follicles obtained via FUE transplantation procedures and combining them with single‐cell sequencing technology, we have delineated the molecular landscape of human scalp hair follicles with unprecedented resolution. Through comparative analysis of follicles from non‐balding and balding regions, we identified key molecular alterations potentially responsible for AGA development.

Consistent with observations in mice [24], human inner hair follicle cells are characterized by the expression of MSX2 as a representative marker, with all matrix lineage cells derived from hair matrix cells expressing this protein [29]. We identified a population of transit‐amplifying cells (TACs) situated developmentally between hair matrix cells and the structurally differentiated cells of the inner follicle layers. This finding supports the model that inner hair follicle morphogenesis is driven by matrix progenitor cells [30]. Interestingly, these hair matrix cells already exhibit transcriptional priming toward differentiation, aligning with previous reports that the spatial positioning of germ layer cells influences lineage commitment. This suggests the existence of a complex and potentially early deterministic process governing cell fate specification within the germ layer.

SFRP1 was identified as a highly specific marker for human outer hair follicle cells. It's notable that BARX2, a well‐established marker for outer layer cells in murine skin, was also highly expressed in human outer follicle cells, indicating evolutionary conservation of follicular cell identity across species, despite the more complex stratification of the outer layers in human hair follicles compared to mice [24]. In our samples, outer layer cells were primarily classified as HFSC‐derived ORS cells and a distinct KRT75^+^ companion layer population. Similar to hair matrix cells in the anagen bulb, HFSCs first transit into a transit‐amplifying cell stage before differentiating into various ORS sub‐lineages, including two topologically distinct branches: suprabasal ORS (sbORS) and basal ORS (bORS). The independent identity of the companion layer is consistent with its developmental origin: during anagen expansion, the companion layer arises from progenitors juxtaposed to the dermal papilla, indicating a cellular origin distinct from that of the ORS. Cell chat analysis revealed that the primary differences between balding and non‐balding hair follicles involved KRT6B^+^ sbORS cells, which mediate structural keratinization and support, and BG cells with canonical HFSC characteristics. These findings suggest that beyond HFSCs, additional cell populations may represent promising new therapeutic targets for AGA treatment.

WNT5A, whose expression is significantly suppressed in HFSCs of balding hair follicles, belongs to the Wnt protein family and primarily activates non‐canonical Wnt signaling pathways (e.g., Ca^2^^+^ signaling and planar cell polarity pathways) [31, 32]. It plays critical roles in embryonic development, cell polarity, migration, and differentiation [33, 34, 35]. For instance, studies in mammalian ear wound repair models have identified WNT5A as a key conserved signal initiating regenerative responses and blastema formation [36]. The role of WNT5A in hair growth and development has also been preliminarily explored. During embryonic hair follicle morphogenesis, it participates in regulating follicle patterning and organization, exhibiting a time‐dependent expression pattern postnatally [18, 37, 38]. However, its function in the cyclic growth of mature hair follicles remains incompletely understood. Some studies have shown that adenovirus‐mediated overexpression of WNT5A in skin tissues inhibits the transition from telogen to anagen, and application of recombinant WNT5A protein also suppresses hair follicle growth [20, 39]. In contrast, our study demonstrated that treatment with Foxy‐5, a WNT5A mimetic peptide, successfully reversed hair growth inhibition in AGA model mice. This apparent discrepancy may be attributed to differences in pathological context and mode of WNT5A pathway activation. Foxy‐5 treatment does not interfere with β‐catenin‐mediated canonical Wnt signaling, whereas recombinant WNT5A may occupy Frizzled receptor binding sites and potentially inhibit canonical Wnt signaling, leading to hair growth suppression. Moreover, the pro‐regenerative effects of WNT5A agonist were more pronounced in mice under androgen‐induced pathological stress than in untreated controls, suggesting a critical crosstalk between AGA pathophysiology and WNT5A signaling. Most importantly, this effect should not be interpreted as direct activation of canonical Wnt/β‐catenin‐driven anagen entry, but rather as modulation of HFSC signaling states via non‐canonical Wnt pathways under androgenic stress.

Recent lineage‐tracing studies have demonstrated that HFSCs require a ‘Wnt‐low’ signaling state to maintain stemness and avoid exhaustion. In this context, WNT5A‐mediated ncWnt signaling may contribute to preserving such a balanced signaling environment rather than directly driving excessive proliferation. In this study, we observed markedly decreased WNT5A expression in HFSCs derived from anagen frontal hair follicles of patients with AGA compared with HFSCs isolated from occipital anagen follicles. In addition, WNT5A protein exhibited cyclic dynamic distribution across the hair cycle, with the strongest immunostaining detected in anagen follicles and gradually attenuated signals during catagen and telogen [40]. Previous studies suggested that WNT5A may sustain the differentiation potential of HFSCs via the CSL–Wnt5a–FoxN1 regulatory cascade, and its reduced expression might contribute to premature catagen entry of hair follicles [41]. DHT potentially suppresses WNT5A secretion by dermal papilla cells within lesional scalp regions, thereby indirectly reducing intrinsic WNT5A levels in HFSCs; relatively low expression of androgen‐metabolizing molecules in occipital follicles may alleviate this inhibitory effect and maintain comparatively higher WNT5A abundance [42]. Downregulated WNT5A in forehead HFSCs may disrupt the homeostasis of canonical and ncWnt signaling, compromise the proliferative and differentiation capacity of HFSCs, and potentially trigger shortened anagen phases and progressive follicular miniaturization. Combined with previous documentation regarding cyclic fluctuations of WNT5A throughout hair cycling, our findings imply that reduced WNT5A expression in forehead HFSCs may serve as a potential molecular mechanism underlying androgen‐driven AGA pathogenesis.

Indeed, previous bulk transcriptomic studies comparing frontal and occipital hair follicles have reported overall downregulation of WNT5A in balding regions, suggesting its potential role as a key anti‐AGA factor, a finding our study further confirms [43]. While conventional research has predominantly focused on the canonical Wnt/β‐catenin signaling pathway [44], our work highlights the importance of the long‐overlooked non‐canonical WNT pathway in AGA pathogenesis, suggesting it may represent a critical direction for future investigations. Interestingly, cell communication analysis revealed that HFSCs act as both primary senders and receivers of WNT5A signals, underscoring their central role in the pathogenic process of AGA.

The significant downregulation of numerous transcription factors in HFSCs of balding hair follicles warrant attention. Our study proposes a novel molecular regulatory axis: WNT5A‐ATF3‐FOSB, whose signaling activity is significantly attenuated in AGA‐affected tissue. Among these, ATF3 is an “adaptive response gene” whose expression is typically low under basal conditions but rapidly induced by various stress signals, such as cellular stress, inflammatory cues, and hormonal changes [45, 46, 47]. Once activated, it participates in regulating diverse processes including cell proliferation, differentiation, and apoptosis [48, 49]. We found that ATF3 expression is also stimulated by WNT5A, and the anti‐AGA effects of WNT5A depend on downstream ATF3 activity, suggesting potential crosstalk between ncWNT signaling and ATF3 regulation that merits further exploration. The functional roles of ATF3 are complex and context‐dependent. For instance, in peripheral DRG sensory neurons, ATF3 promotes nerve regeneration and enhances axonal regrowth after injury [50]. Conversely, elevated ATF3 expression has been observed in various tumor tissues, highlighting the pleiotropic and critical nature of this transcription factor [51, 52, 53]. Through CUT&Tag, we demonstrated that ATF3 in HFSCs likely governs their functional state and differentiation trajectories, as well as modulates broad intracellular signaling pathways. The specificity of ATF3 expression in HFSCs further underscores its central role in hair regeneration and positions it as a promising therapeutic target for future AGA treatments.

As a downstream effector within this molecular axis, FOSB enables HFSCs to unleash greater regenerative potential during the anagen phase. The Fos gene family (including c‐*Fos*, *FosB*, etc.) constitutes a critical component of the AP‐1 transcription factor complex [54, 55]. The role of c‐*Fos* in hair growth has been partially investigated. For instance, maternally derived melatonin promotes offspring hair follicle development by upregulating AP‐1 complex levels, particularly c‐*Fos* and c‐*Jun* expression [56]. Donet et al. demonstrated that glucocorticoid receptor overexpression suppresses hair follicle cell proliferation by downregulating c‐*Fos* gene expression [57]. In contrast, the function of *Fosb* in hair growth remains poorly explored.

Through in vivo knockdown and overexpression experiments, we confirmed that FOSB similarly serves as a key regulator supporting HFSC regenerative capacity during the anagen phase. The absence of significant hair regeneration impairment upon *Fosb* knockdown alone may be attributed to functional compensation by other members of the Fos family. Employing CUT&Tag and dual‐luciferase reporter assays, we validated that ATF3 promotes the transcription and expression of *Fosb*. Notably, reduced expression levels of both ATF3 and FOSB in AGA‐affected regions had been previously found in a bulk transcriptomic study of scalp hair follicles but without further investigation [43]. Our work not only confirms this phenomenon but further elucidates the regulatory relationship between them. Interestingly, both ATF3 and FOSB can participate in forming the AP‐1 complex to regulate the transcription of multiple downstream genes, although the primary constituents of this dimeric transcription factor are typically members of the Jun family (e.g., c‐Jun, JunB, JunD) and the Fos family (e.g., c‐Fos, FosB, Fra‐1, Fra‐2) [58]. We hypothesize that the relationship between ATF3 and FOSB may extend beyond a simple regulator‐target dynamic, potentially involving broader interactions such as mutual transcriptional and translational regulation, as well as functional cooperativity. This also suggests that the role of the AP‐1 complex in AGA pathogenesis warrants further investigation.

Emerging evidence suggests that HFSC activity is tightly regulated by signals from the surrounding immune and stromal microenvironment, which together constitute a dynamic niche controlling hair cycle progression. In this context, Wnt signaling has been recognized as a central mediator of epithelial‐mesenchymal communication within hair follicles and is essential for coordinating stem cell activation and tissue regeneration [59, 60]. Notably, non‐canonical WNT5A signaling has been implicated in regulating stromal‐epithelial interactions and inflammatory responses, suggesting a context‐dependent role in tissue homeostasis [61, 62]. WNT5A‐ATF3‐FOSB axis may therefore represent a functional interface linking niche‐derived cues to intrinsic HFSC transcriptional programs. We speculate that stromal or immune‐derived WNT5A signals could modulate HFSC activity through ATF3‐mediated stress‐responsive transcriptional reprogramming, converging on FOSB‐associated regenerative outputs. Disruption of this axis in androgenetic alopecia may thus reflect both intrinsic HFSC dysfunction and impaired microenvironmental signaling, ultimately contributing to reduced hair follicle regenerative capacity.

This study has several limitations. Although our study provides a high‐resolution transcriptional characterization of human hair follicles in AGA, the limited patient cohort prevents the dataset from representing a comprehensive cellular atlas of the disease. It's mainly due to the availability of paired human scalp specimens from clinical sampling. But enough HFs collection was permitted to make sure the scRNA‐seq results is believable. Future studies incorporating larger patient cohorts and optimized sampling strategies will be required to establish a more complete AGA cell atlas. Moreover, A notable limitation of our single‐cell atlas is the lack of a comprehensive transcriptional profile of the dermal papilla cells (DPC). This omission stems primarily from our use of FUE‐derived samples, a clinically relevant approach that excels at preserving the integrity of the follicular epithelium and its stem cell niche but often fails to efficiently capture the deeply embedded DPC. It limits a complete assessment of epithelial–mesenchymal interactions within the hair follicle niche. Although the total number of fibroblasts or fibroblast‐like cells didn't count for much in all cells, further study still need to level up the number of samples. While the anti‐AGA role of the WNT5A‐ATF3‐FOSB axis has been preliminarily confirmed, the absence of in vivo experiments using HFSC‐specific knockout mice limits the depth of our mechanistic exploration. Furthermore, the intermediate processes through which WNT5A upregulates ATF3 expression remain unclear. For instance, whether WNT5A induces ATF3 expression via Ca^2^^+^‐mediated stress signaling represents a promising regulatory mechanism that requires further investigation to more precisely target the pathogenesis and advance AGA treatment strategies. Most critically, transcription factors such as ATF3 and FOSB are implicated in the development of various cancers. Their activation within hair follicles necessitates careful regulation, highlighting that the translational potential of this molecular axis for clinical AGA intervention remains distant. Additional studies are essential to rigorously evaluate its safety and efficacy before considering therapeutic applications.

In summary, our findings reveal a novel molecular mechanism potentially underlying AGA pathogenesis: the silencing of the WNT5A‐ATF3‐FOSB axis contributes to disease progression. Restoration of this axis is associated with resistance to AGA progression.

## 4 Materials and Methods

### 4.1 Patients

All human subject studies were conducted with appropriate institutional ethical approval. Research involving single‐cell RNA sequencing and histological analysis of human scalp hair follicles was approved by the Ethics Committee of Tongji University Affiliated East Hospital (No. 2025YS‐099) and complied with the 1975 Declaration of Helsinki, as revised in 1983. Written informed consent was obtained from all participants prior to sample collection.

Human hair follicle samples were obtained from discarded cosmetic surgery specimens of healthy individuals who provided written informed consent. Anagen‐phase hair follicles (HFs) were collected from male AGA patients undergoing hair transplantation via follicular unit extraction (FUE), and hair cycle stages were confirmed morphologically using hematoxylin and eosin (H&E) staining. Most of the samples used in the experiments are in anagen. Samples for single‐cell sequencing were obtained from the frontal and occipital regions of three adult male patients aged 25, 30, and 39 years, all clinically diagnosed with androgenetic alopecia. None of the subjects had a history of treatment with finasteride or minoxidil.

### 4.2 Animal Experiments

#### 4.2.1 Mouse Housing Conditions

All experiments utilized adult male mice aged 6 weeks at the start of the study. Mice were obtained from the Experimental Animal Center of Tongji University. Age‐matched littermates were used, and unless otherwise specified, experiments were conducted in parallel. Mice were housed in a specialized animal facility under specific pathogen‐free (SPF) conditions at 22°C, 40%‐70% humidity, and a 12/12‐hour light/dark cycle. All procedures were approved by the Ethics Committee of the Experimental Animal Center of Tongji University (No. TJBB08825101).

#### 4.2.2 AGA Mouse Model

For the AGA mouse model, 6‐week‐old mice received daily intraperitoneal injections of DHT (10 mg/kg, dissolved in corn oil, D‐073, Sigma–Aldrich) at a volume of 0.1 mL for one week to maintain sustained high systemic DHT levels. Injections were continued throughout the experiment until the endpoint.

#### 4.2.3 Foxy‐5 and ATF3 Inducer 1 Treatment Experiments

To activate WNT5A signaling, the validated WNT5A agonist Foxy‐5 (HY‐P1416, MedChemExpress) was used to avoid potential competitive inhibition of the canonical Wnt pathway by recombinant WNT5A protein. Foxy‐5 was dissolved in PBS and administered via subcutaneous injection (1 mg/kg body weight/day). For validation of WNT5A signaling activation, five mice per group were used initially; subsequent groups consisted of three mice each. To enhance ATF3 expression, ATF3 inducer 1 was purchased and administered via daily subcutaneous injections.

#### 4.2.4 Adenovirus‐Mediated Gene Modulation

Adenoviruses were commercially sourced (Viraltherapy Technologies, China). Linearized recombinant Adeno‐X viral DNA was transfected into HEK293 cells (CL‐0001, ProCell System, China) using Lipofectamine 2000 (Invitrogen, Japan). Harvested recombinant adenoviruses were amplified and titrated in HEK293 cells. The final viral titer was adjusted to 1.0 × 10^1^ ^1^ PFU/ml. Custom constructs included Adv‐ZsGreen, Adv‐ZsGreen‐m*Atf3*‐Flag, Adv‐shRNA‐m*Atf3*, Adv‐ZsGreen‐m*Fosb*‐Flag, and Adv‐shRNA‐m*Fosb*. Adenoviruses were administered via multipoint subcutaneous injections: the depilated dorsal area of each mouse was divided into four quadrants, and each quadrant received 10 µl of virus diluted to 1.0 × 10^10^ PFU/ml, resulting in a total volume of 40 µl per mouse. The total viral dose per mouse corresponded to 4.0 × 10^8^ PFU. Injections were performed at 54 and 60 days of age to sustain persistent genetic modulation. This delivery strategy primarily targets dermal and perifollicular compartments, including hair follicle stem cell niches. Adenoviral‐mediated gene expression typically persists for several days to weeks in skin tissue, covering the relevant hair cycle window.

#### 4.2.5 Quantification of Mouse Hair Regrowth

Hair regrowth was quantitatively assessed based on the area covered by newly emerged hair shafts rather than changes in dorsal skin pigmentation. At each indicated time point, standardized photographs of the shaved dorsal skin were acquired. The regions showing visible hair shaft emergence were manually delineated using image analysis software by an investigator blinded to the treatment groups. The hair‐covered area was then calculated and expressed as a percentage of the total shaved dorsal area.

Notably, dorsal skin pigmentation or darkening, which may result from melanocyte activation or environmental lighting conditions, was not used as a criterion for hair cycle staging or regrowth evaluation, as pigmentation does not necessarily reflect functional entry into the anagen phase.

### 4.3 Isolation and Culture of Hair Follicle Stem Cells

Human hair follicles were obtained using follicular unit extraction (FUE) technology. The hair bulbs were removed, and the remaining follicular tissue was digested in PBS containing 4.8 U/mL Dispase II (#7913, STEMCELL Technologies) and 100 U/mL Collagenase IV (#LS004189, Worthington) at 37°C for 60 min. The bulge region, located between the isthmus and the upper hair bulb, was carefully dissected and further treated with Dispase II for 30 min. The dermal sheath was then removed under stereomicroscopic guidance. The remaining epidermal follicular components were digested with 0.025% trypsin (Gibco, Gaithersburg, MD, USA) at 37°C for 10 min.

Digestion was terminated by adding an equal volume of 10% fetal bovine serum (Gibco), and the sample was filtered through a 70 µm strainer (Corning, NY, USA). After centrifugation at 300 × g for 5 min, the cell pellet was resuspended and seeded into six‐well plates pre‐coated with 10 µg/mL human fibronectin (Sigma–Aldrich, St. Louis, MO, USA). Cells were cultured in defined keratinocyte serum‐free medium (K‐SFM, Gibco). A differential enrichment protocol was employed to purify HFSCs from the mixed population of outer root sheath cells. Finally, cells were expanded under standard conditions (5% CO2, 37°C). When cultures reached approximately 70% confluence, cells were detached using 0.025% trypsin, centrifuged at 300 × g for 5 min, and the pellet was resuspended in K‐SFM for subsequent passaging or experimental use.

To activate WNT5A signaling, HFSCs were cultured with 100 µM of Foxy‐5 for 24 h according to previous studies [63]. 30 µM of ATF3 (diluted in 0.5% DMSO) inducer 1 was used to activate ATF3 according to previous studies [64].

### 4.4 Western Blot

HFSCs were lysed in RIPA lysis buffer (Product No. 89900, Invitrogen) containing protease inhibitors (No.78442, Thermo Fisher Scientific). The lysates were heated at 99.9°C for 10 min to complete the protein sample preparation. The protein samples were then separated by SDS‐polyacrylamide gel electrophoresis (SDS‐PAGE) and transferred onto a polyvinylidene fluoride (PVDF) membrane (IPVH00010, Millipore). The membrane was blocked with 5% skim milk (prepared in Tris‐buffered saline with 0.1% Tween 20) at room temperature for 1 h, followed by incubation with primary antibodies overnight at 4°C. The next day, the membrane was incubated with secondary antibodies (goat anti‐rabbit IgG or goat anti‐mouse IgG) for 1 h. After washing the membrane three times with TBST, the signals were detected using the Amersham Imager 680 series ultra‐sensitive multi‐functional imager (Cytiva, USA). Detailed information on the antibodies and their usage in this study is provided in the supplementary materials.

### 4.5 RNA Extraction and RT‐qPCR

HFSCs in the culture dish were washed three times with PBS on ice, then lysed and resuspended in TRIzol (Takara, Japan) (500 µL per well in a six‐well plate). For every 500 µL of the solution, 100 µL of chloroform was added, followed by centrifugation at 12000 rpm for 5 min in a 4°C centrifuge. The upper phase (200 µL) was carefully transferred to a new tube, and 200 µL of isopropanol was added. After standing at room temperature for 10 min, the mixture was centrifuged again. The supernatant was discarded, and the pellet was washed with 75% ethanol and centrifuged. After discarding the supernatant and allowing the pellet to dry, 50 µL of DEPC‐treated water was added to dissolve the RNA. The concentration of RNA was measured using a NanoDrop 2000 spectrophotometer (Thermo Fisher Scientific, USA). Total RNA was reverse transcribed into cDNA using the RevertAid First Strand cDNA Synthesis Kit (Takara, Japan) according to the manufacturer's instructions. RT‐qPCR was performed using SYBR Green Master Mix (Takara, Japan) on a StepOnePlus Real‐Time PCR System (Thermo Fisher Scientific, USA).

### 4.6 Histological Analysis and Immunofluorescence Staining

For histological evaluation, skin tissues were fixed in 4% paraformaldehyde, embedded in paraffin, sectioned at 4 µm thickness, and stained with hematoxylin and eosin (H&E). To assess hair growth, three sections per paraffin block were selected for qualitative and quantitative analysis. Subcutaneous layer thickness was measured as the average distance from the epidermal basement membrane to the base of the hair bulb across multiple fields of view. Hair bulb size was quantified by calculating the average maximum diameter of all visible hair bulbs per section [65]. Measurements were performed using the length measurement or cell counting tools in the open‐source software ImageJ.

For immunohistochemical analysis, paraffin‐embedded tissue sections were deparaffinized in xylene and rehydrated through a graded ethanol series. Antigen retrieval was performed using EDTA buffer (pH 8.0) under microwave heating. Endogenous peroxidase activity was quenched with 3% H_2_O_2_ treatment at room temperature for 25 min, followed by blocking with 3% BSA for 1 h. Sections were incubated with primary antibodies diluted in PBS or blocking buffer at 4°C overnight. After washing, corresponding horseradish peroxidase (HRP)‐conjugated secondary antibodies were applied and incubated at room temperature for 50 min. Tyramide signal amplification (TSA) was then performed using Cy3‐ or FITC‐conjugated tyramide reagents (diluted 1:500 in amplification buffer containing 0.003% H_2_O_2_) for 10 min in the dark at room temperature. For sequential double labeling, bound antibodies were removed after the first TSA cycle by microwave heating in antigen retrieval buffer. The procedure was repeated with a second primary antibody and corresponding FITC or Cy3‐TSA. Nuclei were counterstained with DAPI, and sections were mounted with aqueous anti‐fade mounting medium. Imaging was performed using a fluorescence microscope equipped with appropriate filter sets for DAPI, FITC, and Cy3. Detailed information regarding the antibodies used is provided in the Supplementary Materials.

Immunofluorescence images were analyzed using a standardized quantification workflow. Briefly, all images were acquired under identical microscope settings within each staining batch to ensure comparability. For each marker, at least 3 randomly selected fields per sample were used for quantification. Fluorescence intensity was measured using ImageJ software by defining regions of interest (ROIs) within the hair follicle bulge or other specified compartments based on DAPI nuclear staining and morphological landmarks. Background fluorescence was subtracted using adjacent non‐stained regions. For nuclear markers, only DAPI‐positive nuclei co‐localized signal was included in the analysis. Mean fluorescence intensity (MFI) was calculated per ROI and averaged across multiple fields to represent each biological replicate. Cell counting (where applicable) was performed using threshold‐based segmentation and confirmed manually to avoid double counting. All quantifications were performed in a blinded manner. Quantification criteria were kept consistent across all experimental groups to ensure unbiased comparison.

### 4.7 Transcription Factor Binding Site Prediction

The nucleotide sequence spanning from −2000 bp to +100 bp relative to the transcription start site of the human *FOSB* gene (GenBank accession: NC_000019.10, region: 45465996–45468096; Homo sapiens chromosome 19, GRCh38.p14 Primary Assembly) was retrieved from the NCBI database. Prediction of ATF3 binding sites was performed using the JASPAR database with the ATF3 position weight matrix (PWM). The relative profile score threshold was set to 85%, and binding sites with a prediction score >10 were considered high‐confidence ATF3 binding sites. The same analytical workflow was applied to the corresponding genomic region of the mouse *Fosb* gene.

### 4.8 Cleavage Under Targets and Tagmentation (CUT&Tag)

A total of 1 × 10^5^ hair follicle stem cells (HFSCs) with viability >80% were collected and centrifuged at 600 × g for 3 min at room temperature. The supernatant was discarded, and the cell pellet was washed twice with 100 µL of ice‐cold Cell Wash Buffer (1× PBS supplemented with protease inhibitors). After removing the supernatant, the cells were resuspended in 10 µL of ConA‐coated magnetic beads pre‐equilibrated with ConA Bind Buffer and incubated on a rotator at room temperature for 10 min to facilitate cell capture. The buffer was then removed, and 100 µL of Antibody Buffer containing 0.01% digitonin was added for a 10‐minute incubation at room temperature to block non‐specific binding. After blocking, the buffer was discarded, and 50 µL of primary anti‐ATF3 antibody diluted 1:100 in Antibody Buffer was added, followed by incubation on a rotator at 4°C for 2 h. The primary antibody was removed, and the cells were quickly washed once with 1 mL of Wash Buffer (containing 0.01% digitonin), followed by two additional 5‐minute washes with 0.5 mL of the same buffer under rotation.

After discarding the wash buffer, 50 µL of Protein A/G‐Tn5 transposase complex diluted 1:100 in Antibody Buffer was added, and the sample was incubated on a rotator at room temperature for 1 h. Post‐incubation, the cells were washed three times with Wash Buffer as described above to thoroughly remove unbound complexes.

The wash buffer was completely removed, and 100 µL of Tagmentation Buffer containing 10 mM MgCl_2_ was added to activate Tn5‐mediated DNA cleavage and adapter ligation during a 1‐hour incubation on a horizontal rotator at room temperature. The reaction was terminated by adding 10 µL of 0.5 M EDTA, 11 µL of 10% SDS, and 2.5 µL of 20 mg/mL Proteinase K, followed by digestion at 55°C for 30 min. The digested product was purified using 1.2× volume (approximately 145 µL) of AMPure XP beads and eluted in 21 µL of Elution Buffer. A total of 20 µL of the purified product was combined with 25 µL of 2× high‐fidelity PCR master mix and 2.5 µL of 10 µM Illumina dual‐indexed primers, followed by 15 cycles of PCR amplification. The resulting library was quality‐checked using an Agilent 2100 Bioanalyzer and subjected to sequencing.

### 4.9 ChIP ‐PCR

For ChIP primer design, a 150‐bp sequence flanking the JASPAR‐predicted ATF3 binding site “GGTGACGTAAGC” within the *FOSB* promoter region was selected as the amplification target. The sequence used was: 5′‐CTTGCTGGACAGTGCGGGACTCGATTTGGCGGGGCCGGAGATTTGGGGAAGTTTGTCCAGCAAGGGGCGGGTGACGTAAGCAGGGGGGCGGGTCCCGGGCATATAAATACAGGCTGGCGGGTCTGTGCTTCATTCATAAGACTCAGAGCT‐3′. Primers were designed using Primer3Plus and validated via Primer‐BLAST. The upstream primer sequence was “GACAGTGCGGGACTCGATTT”, and the downstream primer was “GACCCGCCAGCCTGTATTTA”. Both primers exhibited a self‐complementarity value of 5.00 and a self‐3′ complementarity value of 2.00, meeting specificity and stability requirements. The ChIP assay was performed as previously described with minor modifications. Briefly, HFSCs were scraped from culture dishes using phosphate‐buffered saline and cross‐linked with 1% formaldehyde for 8 min, followed by quenching with 1.25 mM glycine for 5 min. Cells were centrifuged at 1,500 rpm for 5 min, and the pellet was resuspended in ChIP lysis buffer (Santa Cruz Biotechnology, Santa Cruz, CA) to isolate nuclei. DNA fragmentation was achieved using a Branson sonicator (Fisher Scientific, Waltham, MA). Cell lysates were incubated overnight at 4°C on a rotator with a mixture of anti‐FOSB‐specific antibody and protein A/G magnetic beads to form bead‐antibody/chromatin complexes. Target DNA fragments were eluted from the beads using ChIP elution buffer containing proteinase K. After purification, the immunoprecipitated DNA fragments bound to FOSB were quantified by qPCR.

### 4.10 Dual‐Luciferase Reporter Assay

The plasmids used for transfection of HFSCs included the promoter plasmid “h‐FOSB‐promoter,” the TF plasmid “h‐ATF3,” and “pcDNA3.1(+)” (Shanghai Carrier‐Sequence Biotechnology Co., Ltd, Shanghai). The night before transfection, 1 × 10^5^ cells were seeded in each well of a 24‐well plate to ensure a cell confluence of 70–80% at the time of transfection. In 50 µL of serum‐free medium, 0.9 µg of the plasmid was added and gently mixed. The Lipofectamine reagent was mixed evenly, and 2 µL of Lipofectamine was diluted with 50 µL of serum‐free medium, gently mixed, and left at room temperature for 5 min. The diluted plasmid and Lipofectamine reagent were then combined, gently mixed, and left at room temperature for 20 min. The 100 µL plasmid/Lipofectamine complex was added to the wells containing 300 µL of complete medium, and the cell culture plate was gently shaken back and forth. After culturing the cells in a 37°C incubator with 5% CO_2_ for 5–6 h, the transfection medium was aspirated and replaced with fresh medium.

The Luciferase Reaction Substrate in lyophilized form was fully dissolved with Luciferase Reaction Buffer (5 mL + 1 vial) to prepare the Luciferase Reaction Reagent. The Luciferase Reaction Reagent II was prepared by mixing Luciferase Reaction Substrate II with Luciferase Reaction Buffer II at a ratio of 1:50. The 5× Cell Lysis Buffer was mixed with ddH_2_O at a ratio of 1:4 and kept on hand. 48 h after transfection, the medium was aspirated, and the cells were washed twice with PBS. Then, 100 µL of 1× Cell Lysis Buffer was added to each well, and the cells were lysed thoroughly at room temperature for 10 min. The lysate was transferred to a 1.5 mL centrifuge tube and centrifuged at 4°C and 12000×g for 10 min. The supernatant was collected for measurement in a luminometer. For firefly luciferase activity detection, an appropriate amount of Luciferase Reaction Reagent was equilibrated to room temperature. Then, 20 µL of the sample was mixed with 100 µL of Luciferase Reaction Reagent and the relative light unit (RLU) was measured after mixing. For luciferase activity detection, an appropriate amount of Luciferase Reaction Reagent II was equilibrated to room temperature. Then, 100 µL of Luciferase Reaction Reagent II was added to each tube, vortexed, and the RLU was measured.

### 4.11 Single‐Cell Sequencing

Single‐cell cDNA libraries were prepared using the DNBelab C Series High‐Throughput Single‐Cell RNA Library Preparation Kit according to the manufacturer's instructions. Sequencing was performed on the MGISEQ‐2000RS platform following the standard operating procedures, with real‐time data analysis. All downstream data analyses were conducted using a combination of custom‐written R scripts and published analysis packages, as described in the following sections.

### 4.12 ScRNA‐seq Analysis

#### 4.12.1 Quality Control and Cell Clustering

After processing the fastq data, 10X Genomics data was obtained. Initial quality control was completed based on thresholds set for total mRNA count, gene number, and mitochondrial read fraction. Cells were filtered based on the following criteria: genes detected per cell (nFeature_RNA) between 500 and 5000, unique molecular identifiers per cell (nCount_RNA) between 500 and 40000, and a percentage of mitochondrial reads (percent.mt) below 10%. The Seurat software package was used for integration and clustering analysis. Gene expression data was log‐normalized and regressed against mitochondrial gene expression and total molecules detected to account for cell‐to‐cell variation. In the primary analysis (the process of categorizing the entire cell population into seven major cell clusters), principal component analysis (PCA) was used to perform linear dimensionality reduction on the entire dataset, with highly variable genes as input. A k‐nearest neighbors (kNN) graph was constructed based on Euclidean distances in PCA space, and clustering was performed using the Louvain algorithm. The clustree package was used to determine the optimal clustering resolution. UMAP was used for dataset visualization. In the secondary analysis (analysis of subclusters within the major cell clusters), the same dimensionality reduction, clustering, and visualization strategies were applied. For each major cell type, highly variable genes and principal components were determined separately. UCell scoring was performed using well‐established marker gene sets for major hair follicle cell subtypes, which were curated from key publications. The complete gene lists for all scoring signatures have been provided as Table S2 to ensure full transparency and reproducibility.

#### 4.12.2 Pseudotime Analysis

The developmental lineage analysis of inner and outer layer cells of the hair follicle was completed using the DiffusionMap package and the Monocle package [66]. DiffusionMap identifies cell differentiation trajectories based on intrinsic diffusion‐like kinetics. Combined with Monocle3, it jointly infers the potential developmental order of the inner and outer lineages of HF.

#### 4.12.3 Cell Chat Analysis

CellChat constructs the Cell Communication Database (CellChatDB) by integrating relevant databases and literature [67]. This database includes several sections such as secreted signaling, ECM‐receptor, and cell‐cell contact. Since we are more interested in whether the hair follicle microenvironment plays a role in AGA (Androgenetic Alopecia), we only included outer layer cells of the hair follicle and major cell populations in the microenvironment, such as skin cells and immune cells, in the calculation. By executing the standard workflow, we predicted the main signaling inputs and outputs for each cell type. To identify significant signaling interactions, we used the “computeCommunProbPathway” function and set the threshold at p‐value = 0.05. After calculating the CellChat object, we completed the visualization according to the basic workflow of the package.

#### 4.12.4 Differential Expression Analysis

The `FindAllMarkers` function was utilized to conduct differential expression analysis on HFSCs categorized under the identities “Forehead” and “Occipital.” The criteria for selecting differentially expressed genes (DEGs) were set at a p‐value threshold of less than 0.05, a log2 fold change (log_2_FC) greater than 1.5, and an expression percentage exceeding 0.3 in both cell groups. Subsequently, Gene Set Variation Analysis (GSVA) was performed on the identified DEGs, based on 50 classic pathways, to identify overall transcriptional differences. To explore the core transcription factors involved, GSEA (Gene Set Enrichment Analysis) was applied to the DEGs using the “TFT: transcription factor targets” gene sets from the Molecular Signature Database.

### 4.13 Statistical Analysis

All quantitative data from animal and cell experiments were presented as mean ± standard deviation (SD). For comparisons between two groups, an unpaired two‐tailed Student's t‐test was used. When all pairwise comparisons among more than two groups were required, Tukey's multiple comparisons test was applied. Statistical analyses were conducted using GraphPad Prism 10. For scRNA‐seq analysis, the Wilcoxon rank‐sum test was used to identify differentially expressed genes and pathway scores between groups. All tests were two‐sided, and *p* < 0.05 was considered statistically significant.

## Author Contributions

Conceptualization: K. Fang, R. Luo, Y. Li, M. Lu. Methodology: R. Luo, K. Fang, J. Xiong, C. Zhao. Investigation: R. Luo, K. Fang, Y. Zhu. Visualization: K. Fang, Z. Li. Supervision: B. Wu, Y. Li, H. Jiang, H. Yan. Writing – original draft: K. Fang. Writing – review & editing: R. Luo, H. Yan, Y. Li, H. Jiang. Funding acquisition: Y. Li, H. Jiang.

## Funding

This work was supported by: National Natural Science Foundation of China (No. 82503037, to J. Xiong). National Fund of the Education Ministry (No. 231103242232720, to Y. Li). The Featured Clinical Discipline Project of Shanghai Pudong Fund (No. PWYts2021‐07, to Y. Li). Shanghai East Hospital Clinical Research Plan Fund (No. SEHHH‐2021(KJ)‐0669‐KJB‐485 and No. SEHHH‐2023(KJ)‐0143‐KYB‐111, to Y. Li).

## Conflicts of Interest

The authors declare no conflicts of interest.

## Supporting information




**Supporting File**: advs77109‐sup‐0001‐SuppMat.docx.

## Data Availability

Original codes generated in this study are available on a github website (https://github.com/KeFang2004/ScRNAseq‐of‐AGA). The sequencing data are available through Sequence Read Archive of National Library of Medicine through (Accession number: PRJNA1252963). All data are available in the main text or the supplementary materials.
