# Supplementary material for: Single‐Cell Profiling Reveals a Protective WNT5A‐ATF3‐FOSB Signaling Axis in Hair Follicle Stem Cells During Androgenetic Alopecia
Source: Adv Sci (Weinh). 2026 Aug 12:e77109. Online ahead of print. doi: 10.1002/advs.77109 (PMC13464553; doi:10.1002/advs.77109)
Supplement: Supplementary file 1 [file ADVS-9999-e77109-s001.docx]

Supplementary Materials for

**Single-cell Profiling Reveals a Protective WNT5A–ATF3–FOSB Signaling Axis in Hair Follicle Stem Cells During Androgenetic Alopecia**

Ruiyu Luo and Ke Fang *et al.*

*Corresponding author. Email:

[fang_ke@tongji.edu.cn](mailto:fang_ke@tongji.edu.cn) (Dr. Ke Fang)

wubaojin@fudan.edu.cn (Prof. Baojin Wu),

dosjhi@126.com (Prof. Hua Jiang),

yufeili@tongji.edu.cn (Prof. Yufei Li).

**This PDF file includes:**

Figure S1 to S18

Table S1 to S4

**Other Supplementary Materials for this manuscript include the following:**

Data S1

Fig. S1. Clustering results of cells in hair follicle tissues.

(**A**) Primary clustering result, cells were divided in to seven different clusters. EPI: epithelial cells. HFC: hair follicle cells. ML: melanocytes. IMM: immune cells. VASC: vascular cells. FIB: fibroblasts. SMC: smooth muscle cells.

(**B**-**H**) Representative cell markers for SMC (**B**), ML (**C**), IMM (**D**), HFC (**E**), FIB (**F**), EPI (**G**) and VASC (**H**), displayed by UMAP plots.

(**I**) Secondary clustering atlas. Mac: macrophages. TC: T cells. LC: lymphocytes. SC: spinous cells. BC: basal cells. SbORS: suprabasal outer root sheath cells. GC: granular cells. BG: bulge cells. MC: basal cells in mitosis. CO: cortex cells in hair follicle. UHF: upper hair follicle cells. bORS: basal outer root sheath cells. HMC: hair matrix cells. ML: melanocytes. TAC: (outer layer hair follicle) transit amplifying cells. IRS: inner root sheath cells. EC: endothelial cells. FB： fibroblasts. CP: companion layer cells. SwG: sweat gland cells. CU: Hair cuticle cells. SMC: smooth muscle cells. SbG: Sebaceous gland cells.

(**J**) Dot plot showing markers for cells in different 24 clusters.

Fig. S2. Stacked violin plots of representative cell markers for inner layer hair follicle cells.

(**A**-**F**) Cell markers calculated by FindAllMarkers for CO (cortex cells) (**A**), MED (medulla cells) (**B**), CU (cuticle cells) (**C**), IRS (inner root sheath cells) (**D**), mTAC (matrix transit amplifying cells) (**E**), HMC (hair matrix cells) (**F**).

Fig. S3. Pseudotime analysis of inner layer cells for each sample.

(**A**-**F**) Pseudotime analysis (left: ranked by pseudotime; right: ranked by cell type) of sample O1 (**A**), O2 (**B**), O3 (**C**), F1 (**D**), F2 (**E**), and F3 (**F**).

Fig. S4. Linage analysis of hair matrix cells, which are also known as germ layer cells.

(**A**) Clustering of hair matrix cells. Four clusters were identified.

(**B**) Gene sets scoring of four differentiation direction (CO: cortex cells, MED: medulla cells, CU: cuticle cells, IRS: inner root sheath cells).

(**C**) Cell markers of the four clusters, displayed by UMAP plots.

(**D** and **E**) Diffusion pseudo-time analysis, visualized by 2D (**D**) and 3D (**E**) diffusion map.

(**F**) Pseudotime analysis conducted with monocle 3 method. Hair matrix cells were set as the differentiation start point.

Fig. S5. Spatial localization of different types of outer root sheath cells.

(**A-D**) Immunofluorescence staining against markers for basal outer root sheath cells. Scale bar = 100 μm.

(**E** and **F**) Immunofluorescence staining against markers for supra-basal outer root sheath cells. Scale bar = 100 μm.

Fig. S6. Pseudotime analysis of outer layer cells for each sample.

(**A**-**F**) Pseudotime analysis (left: ranked by pseudotime; right: ranked by cell type) of occipital group (**A**), sample O1 (**B**), O2 (**C**), O3 (**D**), forehead group (**E**), sample F1 (**F**), F2 (**G**), and F3 (**H**).

**Fig. S7. Cell composition in each sample.**

(**A**) Stacked bar plot showing the ratio taken by specific cell type among all samples.

(**B**) Dot plot showing the value of cell type percentage of each sample. The p value was determined by unpaired Students’ t-test.

Fig. S8. Signaling information flow comparison between forehead and occipital hair follicle cells.

(**A**) Inter-cellular signaling information flow displayed by bar plots. Left: relative flow ratio. Right: absolute flow strength.

Fig. S9. Outgoing and incoming signaling strength in outer hair follicle cells.

(**A**) Scatter plots showing signaling strength changes from occipital to forehead region.

Fig. S10. Network plots demonstrating different signaling strength between cell types and hair follicle groups.

(**A-D**) Network plots showing changes in signaling pathways related to cell adhesion, represented by laminin signaling pathway (**A**), collagen signaling pathway (**B**), CD99 signaling pathway (**C**) and tenascin signaling pathway (**D**).

(**E** and **F**) Network plots showing immune signaling changes, represented by MHC-1 (**E**) and MHC-2 signaling pathways (**F**).

(**G-J**) Network plots showing changes in signaling pathways related to cell growth and proliferation, represented by EGF signaling pathway (**G**), FGF signaling pathway (**H**), NOTCH signaling pathway (**I**), WNT signaling pathway (**J**).

Fig. S11. Violin plots of top differential signaling pathways showing the ncWNT weakening in HFSCs.

(**A-H**) Stacked violin plots for top 8 differential signaling pathways found in bar plots of information flow: ANGPTL signaling pathway (**A**), AGRN signaling pathway (**B**), TWEAK signaling pathway (**C**), CD22 signaling pathway (**D**), NCAM signaling pathway (**E**), CX3C signaling pathway (**F**), NPR2 signaling pathway (**G**), ncWNT signaling pathway (**H**).

(**I**) UMAP plot displaying the expression mode of WNT5A in all cells among the two groups.

(**J**) Dot plot showing cell groups which take a main role in WNT5A expression.

Fig. S12. Cell markers for hair follicle stem cells, presenting in staining sections and UMAP plots.

(**A**) Immunofluorescence staining against markers of HFSCs: KRT15, LGR5, and SOX9.

(**B**) *LMCD1*, *MGST1* and *KRT7* were identified as representative marker genes for clusters of HFSCs.

Fig. S13. WNT5A activation promoted hair growth and matrix cells proliferation.

(**A** and **B**) Quantification of subepidermal layer thickness (**A**) and hair bulb diameter (**B**) after Foxy-5 treatment.

(**C** and **D**) Immunofluorescence staining against Ki67 (**C**) and the quantification (**D**) revealing the enhancement of HFSC linage cells proliferation after WNT5A activation.

ns: no significance, ****: p < 0.0001, determined by one-way ANOVA test.

**Fig. S14. Block of WNT5A signal inhibited hair regrowth under the DHT challenge.**

(**A**) Pictures in time sequence of dorsal mice skin and the quantification of hair regrowth region, showing the different regrowth level after WNT5A blocking (n = 3 per group).

(**B-D**) H&E staining of skin sections (**B**) and the quantification of subepidermal layer thickness (**C**) and hair bulb diameter (**D**) showing the status of hair growth (n = 3, 3 views for quantification per sample).

ns: no significance, *p < 0.05, **: p < 0.01, ***: p < 0.001, ****: p < 0.0001, determined by unpaired Students’ t-test (for **Fig. S14A**) and one-way ANOVA test (for **Fig. S14C** and **D**).

Fig. S15. Rescue experiments further proved the axis combined with WNT5A, ATF3 and FOSB.

(**A**) IF staining against FOSB showed that Foxy-5 elevated the level of FOSB, which could be inhibited by the knock-down of ATF3.

(**B**) Blocking ATF3 expression repressed the effect of Foxy-5 at transcription level.

(**C**) Western blot of FOSB and its quantification revealing that blocking ATF3 expression repressed the effect of Foxy-5 at translation level.

*: p < 0.05, ****: p < 0.0001, determined by one-way ANOVA test.

Fig. S16. *Atf3* knock-down inhibits hair regrowth in mice.

(**A** and **B**) Pictures in time sequence of dorsal mice skin (**A**) and the quantification of hair regrowth region(**B**) demonstrating lowed down speed of hair growth in *Atf3* knock-down mice. *: p < 0.05, **: p < 0.01, determined by unpaired Students’ t-test.

Fig. S17. Hair growth promoting effect of WNT5A is dependent on ATF3.

(**A** and **B**) Pictures in time sequence of dorsal mice skin (**A**) and the quantification of hair regrowth region (**B**), showing disability of WNT5A function after *Atf3* knock-down.

(**C-E**) H&E staining of skin sections (**C**) and the quantification of subepidermal layer thickness (**D**) and hair bulb diameter (**E**) showing the status of hair growth.

ns: no significance, **: p < 0.01, ***: p < 0.001, ****: p < 0.0001, determined by unpaired Students’ t-test (for Fig. S17B) and one-way ANOVA test (for Fig. S17D and E).

Fig. S18. Fosb knock-down further aggregates DHT induced AGA phenotype.

(**A** and **B**) Pictures in time sequence of dorsal mice skin (**A**) and the quantification of the percentage of hair regrowth area (**B**).

ns: no significance, **: p < 0.01, determined by unpaired Students’ t-test.

Table S1. Genes used in cell type annotation

| Cell Type | Marker Genes | Marker Source |
| --- | --- | --- |
| Macrophage (Mac) | *HLA-DQB2* | Classic marker |
| Langerhans cell (LC) | *CD206* | Classic marker |
| T cell (TC) | *CD3D* | Classic marker |
| Spinous layer cell (SC) | *KRT5, KRT10, KRT14* | Ober-Reynolds B, et al. *Nat Genet*. 2023 Aug. |
| Granular layer cell (GC) | *KRT1, KRT10, FLG* | Ober-Reynolds B, et al. *Nat Genet*. 2023 Aug. |
| Basal cell, cluster 1 (BC1) | *KRT5, KRT10, KRT15, POSTN* | Ober-Reynolds B, et al. *Nat Genet*. 2023 Aug. |
| Basal cell, cluster 2 (BC2) | *KRT5, KRT10, KRT15, SAA1* | Ober-Reynolds B, et al. *Nat Genet*. 2023 Aug. |
| Mitosis cell (MC) | *MKI67, KRT10, LHX^-^* | Defined by this work |
| Sebaceous gland cell (SbC) | *MGST1, SCD, ACSBG1* | Classic marker |
| Sweat gland cell (SwC) | *AQP5* | Defined according to the function |
| Upper hair follicle cell (UHF) | *KRT17, KRT79* | Joost S, et al. *Cell Syst*. 2016 Sep; Mesler AL, et al. *Cell Rep*. 2017 Apr. |
| Bulge region cell (BG) | *CD34, CD200, KRT15* | Classic marker |
| Basal outer root sheath cell (bORS) | *KRT5, KRT6A, KRT76, LGR5* | Takahashi R, et al. *J Invest Dermatol*. 2020 Apr. |
| Suprabasal outer root sheath cell (sbORS) | *KRT5, KRT17, KRT33A* | Takahashi R, et al. *J Invest Dermatol*. 2020 Apr. |
| Companion layer cell (CP) | *KRT75* | Takahashi R, et al. *J Invest Dermatol*. 2020 Apr. |
| Transit amplifying cell (TAC) | *KRT14, KRT15, MKI67* | Hsu YC, et al. *Cell*. 2014 May. |
| Hair matrix cell (HMC) | *MSX2, MKI67* | Wu S, et al. *Cell Discov*. 2022 May. |
| Cortex cell (CO) | *MSX2, KRT31* | Wu S, et al. *Cell Discov*. 2022 May. |
| Inner root sheath cell (IRS) | *MSX2, KRT23, KRT73* | Wu S, et al. *Cell Discov*. 2022 May. |
| Cuticle cell (CU) | *MSX2, KRT72* | Wu S, et al. *Cell Discov*. 2022 May. |
| Melanocyte (ML) | *MLANA* | Classic marker |
| Endothelial cell (EC) | *PECAM1, VWF, FLT4* | Classic marker |
| Fibroblast (FB) | *COL1A1, THY1* | Classic marker |
| Smooth muscle cell (SMC) | *ACTA2* | Classic marker |

Table S2. Genes used in UCell analysis

| Cell Type | Marker Genes | Marker Source |
| --- | --- | --- |
| Basal outer root sheath cell (bORS) | *"KRT5","ITGA6","KRT15","MSX2-","KRT6A-","KRT6B-","KRT6C-","KRT17-","KRT35-","KRT85-"* | Takahashi R, et al. *J Invest Dermatol*. 2020 Apr. |
| Suprabasal outer root sheath cell (sbORS) | *"KRT5","KRT6A","KRT6B","KRT6C","KRT17","MSX2-","KRT15-","ITGA6-","KRT35-","KRT85-"* | Takahashi R, et al. *J Invest Dermatol*. 2020 Apr. |
| Companion layer cell (CP) | *"KRT75","CST6","KRT79","KRT6A-","KRT6B-","KRT6C-","KRT17-","MSX2-","KRT15-","ITGA6-","KRT35-","KRT85-* | Takahashi R, et al. *J Invest Dermatol*. 2020 Apr. |
| Cortex cell (CO) | *"MSX2","KRT85","KRT5-","KRT73-","KRT71-","KRT82-","KRT14-"* | Wu S, et al. *Cell Discov*. 2022 May. |
| Inner root sheath cell (IRS) | *"MSX2","KRT73","KRT71","KRT25","KRT28","KRT5-","KRT85-"* | Wu S, et al. *Cell Discov*. 2022 May. |
| Cuticle cell (CU) | *“MSX2”, “KRT72”* | Wu S, et al. *Cell Discov*. 2022 May. |

Table S3. Primers used for the analysis of mRNA expression

| Type | Gene | Forward Primer | Reverse Primer |
| --- | --- | --- | --- |
| Human | *β-Actin* | ATATCGCTGCGCTGGTCGTC | AGGATGGCGTGAGGGAGAGC |
|  | *ATF3* | CGCTGGAATCAGTCACTGTCAG | CTTGTTTCGGCACTTTGCAGCTG |
|  | *FOSB* | TCTGTCTTCGGTGGACTCCTTC | GTTGCACAAGCCACTGGAGGTC |

Table S4. Key resources

| REAGENT or RESOURCE | SOURCE | IDENTIFIER |
| --- | --- | --- |
| Antibodies | | |
| Monoclonal rabbit anti-KRT5 | Proteintech | Cat.# 28506-1-AP; RRID: AB_2881159 |
| Monoclonal rabbit anti-KRT6B | Proteintech | Cat.# 17391-1-AP; RRID: AB_2134318 |
| Monoclonal rabbit anti-KRT7 | Proteintech | Cat.# 22208-1-AP; RRID: AB_2879030 |
| Monoclonal mouse anti-KRT14 | Proteintech | Cat.# 60320-1-Ig; RRID: AB_2881431 |
| Monoclonal mouse anti-KRT15 | Proteintech | Cat.# 60247-1-Ig; RRID: AB_2881369 |
| Ployclonal rabbit anti-KRT82 | Affinity Biosciences | Cat.#DF2557; RRID: AB_2839763 |
| Polyclonal rabbit anti-KRT85 | Affinity Biosciences | Cat.#DF2558; RRID:  AB_2839764 |
| Polyclonal rabbit anti-SFRP1 | Proteintech | Cat.# 26460-1-AP; RRID: AB_2880522 |
| Polyclonal rabbit anti-MSX2 | Proteintech | Cat.# 26199-1-AP; UNIPROT ID: P35548 |
| Polyclonal rabbit anti-TCHH | ABclonal technology | Cat.# A15731; RRID: AB_2763146 |
| Monoclonal mouse anti-GATA3 | Proteintech | Cat.# 66400-1-Ig; RRID: AB_2881774 |
| Monoclonal rabbit anti-LMCD1 | Proteintech | Cat.# 67834-1-Ig; RRID: AB_2918596 |
| Polyclonal rabbit anti-MGST1 | Proteintech | Cat.# 15906-1-AP; UNIPROT ID: P10620 |
| Polyclonal rabbit anti-ITGA6 | Proteintech | Cat.# 27189-1-AP; RRID: AB_2880792 |
| Polyclonal rabbit anti-SERPINF1 | Proteintech | Cat.#26045-1-AP; UNIPROT ID: P36955 |
| Polyclonal rabbit anti-COL4A1 | Proteintech | Cat.# 30850-1-AP; UNIPROT ID:P02462 |
| Polyclonal rabbit anti-TNFRSF19 | MedChemExpress | Cat.# YA2865 |
| Monoclonal rabbit anti-PPARGC1A | Proteintech | Cat.# 66369-1-Ig; RRID: AB_2828002 |
| Polyclonal rabbit anti-PARD3 | Proteintech | Cat.# 11085-1-AP; RRID: AB_2159649 |
| Polyclonal rabbit anti-WNT5A | Proteintech | Cat.# 29793-1-AP; RRID: AB_3086163 |
| Polyclonal rabbit anti-ATF3 | Abcam | Cat.# ab207434; |
| Polyclonal rabbit anti-FOSB | Affinity Biosciences | Cat.# AF5010 |
| Monoclonal mouse anti-GAPDH | Abmart | Cat.#M20006; RRID: AB_2737054 |
| Goat anti-mouse IgG-HRP | Abmart | Cat.#M21001; RRID: AB_2713950 |
| Goat anti-rabbit IgG-HRP | Abmart | Cat.#M21002; RRID: AB_2713951 |
| Bacterial and virus strains | | |
| Ad-ZsGreen-mAtf3-Flag overexpression adenovirus | Viraltherapy Technologies | N/A |
| Ad-ZsGreen-mFosb-Flag overexpression adenovirus | Viraltherapy Technologies | N/A |
| Ad-ZsGreen-shRNA-mAtf3 knock-down adenovirus | Viraltherapy Technologies | N/A |
| Ad-ZsGreen-shRNA-mFosb knock-down adenovirus | Viraltherapy Technologies | N/A |
| Ad-ZsGreen overexpression control adenovirus | Viraltherapy Technologies | N/A |
| Ad-ZsGreen knock-down control adenovirus | Viraltherapy Technologies | N/A |
| Chemicals, peptides, and recombinant proteins | | |
| Foxy-5 | MedChemExpress | Cat.#HY-P1416 |
| ATF3 inducer 1 | MedChemExpress | Cat.# HY-151923 |
| 5α-Dihydrotestosterone (DHT) | Sigma-Aldrich | Cat.#D073 |
| Trizol | Vazyme | Cat.#R401 |
| HiScript II Q RT SuperMix for qPCR | Vazyme | Cat.#R222-01 |
| ChamQ SYBR qPCR Master Mix | Vazyme | Cat.#Q331-02 |
| RIPA Lysis Buffer | Beyotime | Cat.#P0013B |
| Protease and phosphatase inhibitor cocktail | Beyotime | Cat.#P1050 |
| Methanol | Aladdin | Cat.#M116118 |
| Ethanol | Aladdin | Cat.#E111964 |
| Avertin | MeilunBio | Cat.#MA0478 |
| Bleomycin | Beyotime | Cat.#ST1450 |
| Collagenase IV | ThermoFisher Scientific | Cat.#17104019 |
| DNase I | Roche | Cat.#11284932001 |
| Dispase II solution | Beyotime | Cat.#ST2339 |
| DAPI | Sigma | Cat.#D9542 |
| 4% Paraformaldehyde fixative | Biosharp | Cat.#BL539A |
| Xylene | Aladdin | Cat.#X112050 |
| 5% BSA blocking buffer | Solarbio | Cat.#SW3015 |
| Fetal bovine serum | Sigma-Aldrich | Cat.#F8318 |
| DMEM | ThermoFisher Scientific | Cat.#12491015 |
| Critical commercial assays | | |
| SABC-HRP Kit | Beyotime | Cat.#P0603 |
| Total HCY ELISA kit | Finetest | Cat.#EU20003 |
| Hydroxyproline Assay kit (Colorimetric) | Abcam | Cat.#ab222941 |
| Immunoprecipitation kit with Protein A+G Magnetic Beads | Beyotime | Cat.#P2179S |
| Pierce BCA Protein Assay Kit | Thermo Fisher Scientific | Cat.#A55864 |
| Masson's Trichrome Staining Kit | Beyotime | Cat.#C0189 |
| Hematoxylin and Eosin Staining Kit | Beyotime | Cat.#C0105 |
| Deposited data | | |
| Single cell RNA-seq data | This paper | SRA: PRJNA1252963 |
| Experimental models: Organisms/strains | | |
| Mouse: C57BL/6J | Shanghai Model Organisms Center, Inc. | Cat. NO. SM-001 |
| Oligonucleotides | | |
| Primers for qPCR, see Table S1 | This paper | N/A |
| Software and algorithms | | |
| ImageJ | Schneider et al., 2012 | [imagej.net/ij/index.html](https://imagej.net/ij/index.html) |
| Graphpad Prism 9 | GraphPad | [www.graphpad.com/](https://www.graphpad.com/) |
| IBM SPSS Statistics 29.0.1.0 | IBM Corp. | [www.ibm.com/spss](https://www.ibm.com/spss) |
| Megellan | TECAN | [lifesciences.tecan.com/software-magellan](https://lifesciences.tecan.com/software-magellan) |
| cellSens | Olympus | [lifescience.evidentscientific.com.cn/zh/software/cellsens/](https://lifescience.evidentscientific.com.cn/zh/software/cellsens/) |
| Leica LAS X | Leica | N/A |
| R | The R Project for Statistical Computing | [www.r-project.org/](https://www.r-project.org/) |
| DESeq2 | Love et al., 2014 | [bioconductor.org/packages/release/bioc/html/DESeq2.html](https://bioconductor.org/packages/release/bioc/html/DESeq2.html) |
| Seurat | Hao et al., 2021 | [satijalab.org/seurat/](https://satijalab.org/seurat/) |
| Clustree | Zappia et al., 2018 | [github.com/lazappi/clustree](https://github.com/lazappi/clustree) |
| ggplot2 | Wickham et al., 2016 | [ggplot2.tidyverse.org/](https://ggplot2.tidyverse.org/) |
| Monocle3 | Qiu et al., 2017 | <https://bioconductor.org/packages/monocle/> |

Data file S1.

Original data for visualization.

Data file S2.

Cell markers for primary clustering, including seven clusters.

Data file S3.

Cell markers for secondary clustering, including 24 clusters.

Data file S4.

Cell markers for inner layer of hair follicle cells.

Data file S5.

Cell markers for outer layer of hair follicle cells.

Data file S6.

Peak annotation of CUT-Tag.
